# Non-Robertsonian translocations involving chromosomes 13, 14, or 15 in male infertility: 28 cases and a review of the literature

**DOI:** 10.1097/MD.0000000000014730

**Published:** 2019-03-01

**Authors:** Hongguo Zhang, Ruixue Wang, Yang Yu, Haibo Zhu, Leilei Li, Xiao Yang, Xiaonan Hu, Ruizhi Liu

**Affiliations:** a Center for Reproductive Medicine and Center for Prenatal Diagnosis, First Hospital; b Jilin Engineering Research Center for Reproductive Medicine and Genetics, Jilin University, Changchun, China.

**Keywords:** breakpoint, genetic counseling, male infertility, non-Robertsonian translocation

## Abstract

For genetic counseling of male carriers of chromosomal translocations, the specific chromosomes and breakpoints involved in the translocation are relevant to know. The structural chromosomal abnormalities may lead to abnormal sperm counts, infertility, and miscarriage. These are related to the specific chromosomes and breakpoints involved in the translocation. To date, over 200 cases of non-Robertsonian translocation in male carriers have been described that involve chromosomes 13, 14, or 15.

This study reports of 28 male carriers from our clinic with balanced reciprocal translocations of chromosome 13, 14, or 15, and a literature review of 201 cases. The 28 male carriers from our clinic were diagnosed by cytogenetic analyses: 19 subjects suffered from pregestational infertility and 9 from gestational infertility. The most common translocations were t(7;13), t(10;14), and t(3;15), observed respectively in 13 (46%), 8 (29%), and 8 (29%) of our subjects. The literature cases (n = 201) involved chromosome 13 (n = 83, 41%), chromosome 14 (n = 56, 28%) or 15 (n = 62, 31%) in which 75 breakpoints were identified, the most common breakpoint, 13q22, was observed in 12 subjects (6%), followed by 14q32 (n = 11), 15q15 (n = 9), and 15q22 (n = 9). Most breakpoints were related to gestational infertility, while breakpoints at 13p13, 13p12, 13p11.2, 13p11, 13q11, 13q15, 14p12, 14p10, 15p13, 15p10, and 15q22.2 were associated with pregestational infertility.

Carriers of non-Robertsonian translocations involving chromosome 13, 14, or 15 and experiencing infertility should receive counseling with regard to chromosomal breakpoints as there seem to be consequences for treatment. Intracytoplasmic sperm injection with preimplantation genetic diagnosis (PGD) for the carriers with oligozoospermia, microscopic testicular sperm extraction or sperm from the sperm bank for the carriers with azoospermia should be considered for pregestational infertility. The carriers with gestational infertility can choose PGD or prenatal diagnosis

## Abstract 2

Supplemental Digital Content is available in the text

## 1 Introduction

Chromosomal abnormalities play a major role in male infertility as structural chromosomal aberrations are up to 10 times more common.^[1,2]^ Karyotype analysis is therefore relevant in the work-up of infertility.^[3]^ The structural chromosomal abnormalities may lead to abnormal sperm counts, infertility, and miscarriage.^[4,5]^ Robertsonian translocation is one of the most common structural chromosomal abnormalities, and involves group D (chromosomes 13, 14, 15) or G chromosomes (chromosomes 21, 22). Previous research has shown that carriers of Robertsonian translocation exhibit azoospermia because of changes in interchromosomal effect or show an increased frequency of disomic and diploid spermatocytes.^[6,7]^ However, reports of non-Robertson (balanced) translocations involving group D chromosome are rare.

Carriers of balanced translocations are phenotypically not to be recognized; however, they may suffer infertility or spontaneous abortions.^[8]^ These are related to the specific chromosomes and breakpoints involved in the translocation.^[9]^ Previous reports indicate that the involvement of group D chromosomes in non-Robertson translocation is related to male infertility. Mikelsaar et al^[10]^ further a reported an infertility case with balanced reciprocal translocation t(5;13)(q33;q12.1) and a microduplication in the region 9q31.1; they hypothesize that haploinsufficiency of the TUBA3C (tubulin alpha 3c) gene could cause the sperm immobility and abnormal sperm morphology as observed in this case. Jiang et al^[11]^ reported oligospermia in a carrier of the reciprocal translocation of t(8;15) and identified an association between chromosomal behavior and apoptosis of primary spermatocytes. In addition, the KATNAL1 gene that plays a role in the regulation of Sertoli cell microtubule dynamics has been mapped on chromosome 13q12.3, its role in spermiogenesis is indispensable.^[12]^ Previous studies have shown that the chromosome 15q15.3 region harbors CATSPER2, STRC, and PPIP5K1 genes, all associated with severely impaired spermatogenesis,^[13]^ while spermatogenesis-associated protein 8 (SPATA8), a testis-specific gene, has been mapped to chromosome 15q26.2.^[14]^ If translocation breakpoints interrupt these vital gene structures, then it is highly likely that the patients involved will suffer infertility.

The aim of this study is to explore the association between the clinical characteristics of male infertility in carriers of non-Robertsonian translocations involving the chromosomes 13, 14, or 15, with regard to the provision of appropriate genetic counseling.

## 2 Subjects and methods

### 2.1 Subjects and study design

We performed a single-centre retrospective study of subjects with non-Robertsonian translocations in chromosome 13, 14, or 15 in infertile men, and searched the literature using the PubMed database using the search terms, which is “chromosome/translocation/sperm” and “chromosome/translocation/abortion” on June 1 to 15, 2018.

The study was approved by the Ethics Committee of the First Hospital of Jilin University. Between July 2010 and December 2015, 28 men suffering from infertility were recruited from the outpatients department of the Centre for Reproductive Medicine at the First Hospital of Jilin University, Changchun, China. All subjects underwent a physical examination and a semen analysis, and completed a detailed questionnaire on smoking (tobacco), drinking (alcohol), marital status, childbearing history, spontaneous abortion, medical history, and working conditions. According to our previously published classification of smoking and drinking,^[15]^ all questionnaires included smoking profile (733 [14%] heavy smokers, 1937 [37%] moderate, 2251 [43%] mild, and 314 [6] nonsmokers), and alcohol drinking profile (heavy drinking 157 [3%], moderate 942 [18%], mild 3507 [67%], and no alcohol drinking 629 [12%]). Azoospermia and oligozoospermia were defined by criteria described previously.^[16]^

### 2.2 Cytogenetic analysis

From each subject, we carried out a karyotype analysis of peripheral blood lymphocytes: peripheral blood (0.5 mL) was cultured in sterile tubes containing 30 U/mL heparin for 72 hours (Culture media, Yishengjun; Guangzhou Baidi Biotech, Guangzhou, China) and subsequently treated with 20 μg/mL colcemid for 1 hour. G-banding of metaphase chromosomes and karyotype analysis were performed as in our previous study.^[16]^

### 2.3 Translocation breakpoints

We used PubMed to carry out a literature search for non-Robertsonian translocations involving chromosomes 13, 14, or 15 in association with male infertility. We excluded translocations involving chromosome 13, 14, or 15, without reported breakpoints (n = 3). We analyzed the relationship between translocation breakpoints and male infertility and miscarriage.

## 3 Results

We identified in our 28 cases with non-Robertsonian translocations involvement of chromosomes 13 (n = 10), 14 (n = 7), or 15 (n = 11). Nineteen subjects with pregestational infertility (the main characteristic being azoospermia or oligozoospermia), the remaining nine subjects exhibited gestational infertility (the main finding being normal semen parameters, in which the patient's partner conceived but tended to miscarry). The karyotype analyses of these 28 subjects in relation to chromosome 13, 14, or 15 translocations are summarized in Table 1.

**Table 1:**
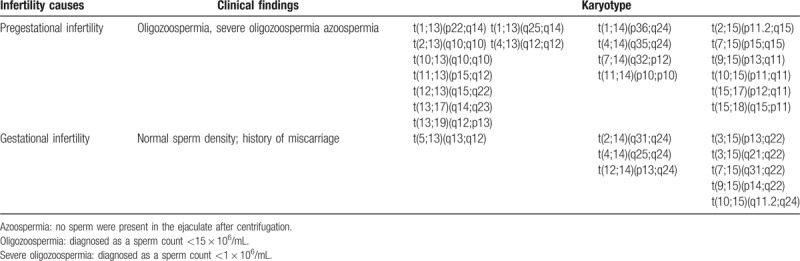
Karyotypes of non-Robertsonian translocation involving group D chromosomes and their clinical features.

**Figure 1:**
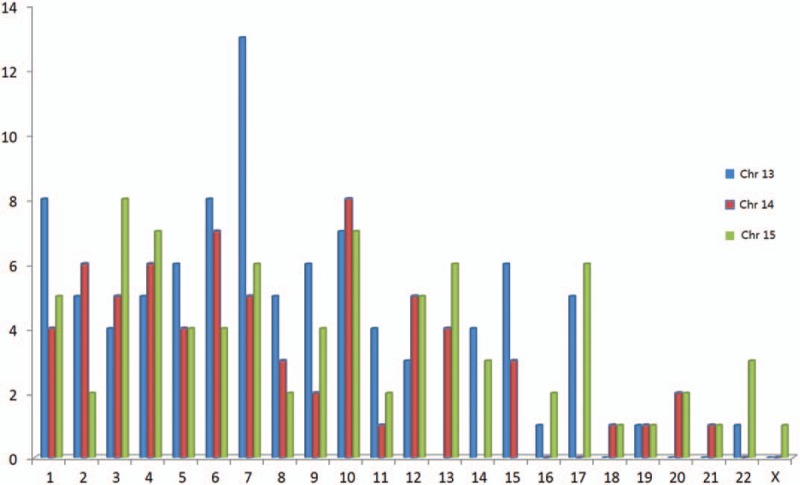
Distribution of chromosomes involved in translocations with chromosome 13, 14, and 15. Chr = chromosome.

Our literature searches identified 201 carriers of non-Robertsonian translocation, 83 subjects in chromosome 13, 56 subjects in chromosome 14 and 62 in chromosome 15. The karyotypes of, and breakpoints in, group D chromosomes, and their related clinical symptoms, are summarized in a supplementary files (Table 1). The most common translocations are t(7;13), t(10;14), and t(3;15), observed respectively in 13, 8, and 8 subjects. In male infertility, the distribution of other chromosomes involved in the translocation with chromosome 13, 14, or 15 are shown in Figure 1.

The literature search identified 75 breakpoints. The most common breakpoint, at 13q22, was observed in 12 subjects, followed by 14q32 (n = 11), 15q15 (n = 9), and 15q22 (n = 9). Most breakpoints are related to gestational infertility, while breakpoints at 13p13, 13p12, 13p11.2, 13p11, 13q11, 13q15, 14p12, 14p10, 15p13, 15p10, and 15q22.2 are associated with pregestational infertility (see supplementary files: Table 2).

## 4 Discussion

Chromosomal abnormality is a major genetic factor contributing to male infertility.^[2]^ Previous studies have reported that the presence of chromosomal translocations can alter the process of spermatogenesis.^[17]^ Indeed, reciprocal and Robertsonian translocations have been shown to lead to male infertility or spontaneous abortion by altered segregation pattern, increased sperm aneuploidy, or altered semen parameters.^[4,6,7,18]^ These effects are associated with specific chromosomes and breakpoints involved in translocation.^[9]^ Balanced reciprocal translocation involving chromosomes 13, 14, or 15 are reported to be closely related to male infertility and recurrent pregnancy loss.^[10,19,20]^ As male infertility is divided into pregestational and gestational infertility,^[21]^ we divided the 28 subjects identified as carriers of balanced reciprocal translocation involving chromosomes 13, 14, or 15, and found 19 of these suffered pregestational infertility, the remaining 9 patients gestational infertility.

We similarly analyzed the literature and identified of the 201 subjects, 83 involving chromosomes 13, 56 involving chromosome 14, and 62 subjects involving chromosome 15. The most common translocations reported are t(7;13), t(10;14), and t(3;15), observed, respectively, in 13, 8, and 8 subjects. The non-Robertsonian translocations involving chromosomes 13, 14, or 15 are at increased risk of infertility or spontaneous abortions. Previous research has shown that abnormal synapsis in translocation carriers could lead to meiotic arrest and influence the spermatogenesis^[19]^ by associated abnormal chromosome behavior with apoptosis in primary spermatocytes.^[11]^

A breakpoint in autosomal translocation may disrupt the genes responsible for spermatogenesis or impair the pairing of synaptic complexes during meiosis, thus resulting in reproductive failure.^[22]^ To investigate the relationship between breakpoints in chromosomes 13, 14, and 15 and male infertility, we carried out an analysis of the related literature and identified a close association between breakpoints in these translocation carriers and male infertility and reproductive failure. In total, 75 breakpoints were identified. Of these, the most common breakpoint, at 13q22, was observed in 12 subjects, followed by 14q32 (n = 11), 15q15 (n = 9) and 15q22 (n = 9). Most breakpoints are related to gestational infertility, while breakpoints at 13p13, 13p12, 13p11.2, 13p11, 13q11, 13q15, 14p12, 14p10, 15p13, 15p10, and 15q22.2 are associated with pregestational infertility. Consequently, we recommend that patients undergoing genetic counseling for balanced translocation carriers should also receive preimplantation genetic diagnosis or prenatal testing.^[23]^ In particular, the carriers of non-Robertsonian translocations involving chromosome 13, 14, or 15. A limitation of this study is the lack of detailed research regarding the specific molecular effects of each translocation by molecular-cytogenetic methods. Therefore, we are unable to explain the relationship between each breakpoint and spermatogenesis.

## 5 Conclusion

Our results show that 28 subjects are identified as carriers of balanced reciprocal translocation involving chromosomes 13, 14, or 15. Nineteen of these have experienced pregestational infertility, while 9 present with gestational infertility. Combined with literature analyses a total of 75 breakpoints are identified. Pregestational infertility is associated more of the chromosome 13 with the breakpoints at 13q14, while gestational infertility with 14q32. These differences have consequences for infertility treatment and genetic counseling. Intracytoplasmic sperm injection with PGD for the carriers with oligozoospermia, microscopic testicular sperm extraction or sperm from the sperm bank for the carriers with azoospermia should be considered for pregestational infertility. The carriers with gestational infertility can choose PGD or prenatal diagnosis.

## Author contributions

HZ, 1st author, case analysis, and writing the article; RW,YY, clinical cases collection and analysis; HZ, 4th author, LL, cytogenetic analysis; XH, literature search; XY, data curation; RL, critical revision of the article; and final approval of article.

**Data curation:** Xiao Yang.

**Funding acquisition:** Ruizhi Liu.

**Investigation:** Ruixue Wang, Yang Yu.

**Methodology:** Haibo Zhu, Leilei Li.

**Software:** Xiaonan Hu.

**Writing – original draft:** Hongguo Zhang.

**Writing – review & editing:** Ruizhi Liu.

## Supplementary Material

Supplemental Digital Content
