# Supplementary material for: Non-Robertsonian translocations involving chromosomes 13, 14, or 15 in male infertility: 28 cases and a review of the literature
Source: Medicine (Baltimore). 2019 Mar 1;98(9):e14730. doi: 10.1097/MD.0000000000014730 (PMC6831198; doi:10.1097/MD.0000000000014730)
Supplement: Supplemental Digital Content [file medi-98-e14730-s001.docx]

Supplementary Table 1. Breakpoints in non-Robertsonian translocation involving group D chromosomes and clinical features, as reported in previous literature

| Cases | Karyotype | Breakpoints | Clinical findings | Reference |
| --- | --- | --- | --- | --- |
| 1 | t(1;13) | 1p22; 13q14 | Oligozoospermia | Li et al., 2012 ^[21]^ |
| 2 | t(1;13) | 1q24; 13q10 | Oligozoospermia | Yakut et al., 2006 ^[49]^ |
| 3 | t(1;13) | 1q41; 13q22 | Normal semen | Perrin et al., 2009 ^[38]^ |
| 4 | t(1;13) | 1q42; 13q14 | Oligozoospermia | Vozdova et al., 2013 ^[23]^ |
| 5 | t(1;13) | 1q41; 13q22 | Repeated reproductive failures | Oliver-Bonet et al., 2002 ^[87]^ |
| 6 | t(1;13) | 1q42.12; 13q32.2 | PGD | Escudero et al.,2003 ^[37]^ |
| 7 | t(2;13) | 2p16; 13q31 | Infertility or PGD | Brugnon et al., 2006 ^[64]^ |
| 8 | t(2;13) | 2q22.2; 13q33.1 | Recurrent pregnancy loss | Sider et al., 1988 ^[47]^ |
| 9 | t(2;13) | 2q32; 13q11 | ICSI | Gekas et al., 2001 ^[69]^ |
| 10 | t(2;13) | 2q35; 13q32 | Repeated miscarriage | Goddijn et al., 2004 ^[62]^ |
| 11 | t(3;13) | 3p21; 13p11.2 | Severe oligozoospermia | Mierla et al., 2014 ^[24]^ |
| 12 | t(3;13) | 3p21; 13p11.2 | Severe oligozoospermia | Mierla et al., 2014 ^[72]^ |
| 13 | t(3;13) | 3p13;13q14 | Recurrent fetal wastage | Fryns et al., 1998 ^[25]^ |
| 14 | t(3;13) | 3q27; 13q11 | Oligospermia | Perrin et al., 2009 ^[38]^ |
| 15 | t(4;13) | 4q21; 13q32 | 4 Miscarriages | Olszewska et al., 2017 ^[54]^ |
| 16 | t(4;13) | 4q12;13q12 | Oligoasthenozoospermia | Li et al., 2012 ^[21]^ |
| 17 | t(4;13) | 4q21.3;13q22 | Recurrent fetal wastage | Fryns et al., 1998 ^[25]^ |
| 18 | t(4;13) | 4q23; 13qter | Multiple abortions | Campana et al., 1986 ^[51]^ |
| 19 | t(5;13) | 5q34; 13q33 | Repeated miscarriages | Iyer et al., 2007 ^[73]^ |
| 20 | t(5;13) | 5p13; 13q34 | Fetal abnormalities | Zhang et al., 2006 ^[45]^ |
| 21 | t(5;13) | 5q21; 13q12.1 | Miscarriage | Ikuma et al., 2015 ^[80]^ |
| 22 | t(5;13) | 5q11; 13q33 | Spontaneous abortions | Pellestor et al., 1989 ^[55]^ |
| 23 | t(5;13) | 5q15; 13p12 | Oligozoospermia | Matsuda et al., 1992 ^[41]^ |
| 24 | t(6;13) | 6p21.3; 13q14.3 | PGD | Zhang et al., 2014 ^[39]^ |
| 25 | t(6;13) | 6p21.1; 13q32 | PGD | Yakut et al., 2006 ^[49]^ |
| 26 | t(6;13) | 6p22.2; 13q14.2 | PGD | Kyu Lim et al.,2004 ^[46]^ |
| 27 | t(6;13) | 6p22.2; 13q14.13 | PGD | Ko et al.,2010 ^[50]^ |
| 28 | t(6;13) | 6q10; 13q10 | Spontaneous abortion | Stephenson et al., 2006 ^[63]^ |
| 29 | t(6;13) | 6q13; 13q22 | Spontaneous abortion | Bourrouillou et al., 1986 ^[60]^ |
| 30 | t(6;13) | 6q25.1; 13q31 | Prior miscarriage and PGD | Ikuma et al., 2015 ^[80]^ |
| 31 | t(6;13) | 6q21; 13q21 | Normozoospermia | Haapaniemi Kouru et al., 2017 ^[40]^ |
| 32 | t(7;13) | 7p13; 13q21.2 | Recurrent pregnancy loss | [Kochhar](http://www.ncbi.nlm.nih.gov/pubmed/?term=Kochhar%20PK%5BAuthor%5D&cauthor=true&cauthor_uid=22672580) et al., 2013 ^[26]^ |
| 33 | t(7;13) | 7q11.22; 13q21.3 | Primary infertility 6 years | Vozdova et al., 2013 ^[23]^ |
| 34 | t(7;13) | 7q35;13q13 | Infertility | Gada Saxena et al., 2012 ^[27]^ |
| 35 | t(7;13) | 7p21; 13q21.1 | PGD | Kyu Lim et al., 2004^[46]^ |
| 36 | t(7;13) | 7p15; 13q15 | Oligozoospermia | Zhang et al., 2015 ^[28]^ |
| 37 | t(7;13) | 7q22; 13q24 | Repeated spontaneous abortions | Niroumanesh et al., 2011^[29]^ |
| 38 | t(7;13) | 7q31;13q31 | Recurrent fetal wastage | Fryns et al., 1998 ^[25]^ |
| 39 | t(7;13) | 7p15;13q33 | Recurrent fetal wastage | Fryns et al., 1998 ^[25]^ |
| 40 | t(7;13) | 7q22; 13q34 | Azoospermia | Poli et al., 2016 ^[30]^ |
| 41 | t(7;13) | 7q34; 13q13 | 2 early miscarriages | Midro et al., 2006 ^[48]^ |
| 42 | t(7;13) | 7p13; 13q21.2 | Spontaneous abortion | Stephenson et al., 2006 ^[63]^ |
| 43 | t(7;13) | 7p13; 13q14.2 | PGD | Ko et al.,2010 ^[50]^ |
| 44 | t(7;13) | 7q22; 13q12 | PGD | Findikli et al., 2003 ^[67]^ |
| 45 | t(8;13) | 8p22; 13q13 | Oligoashenoteratozoospermia | Pellestor et al., 2001 ^[86]^ |
| 46 | t(8;13) | 8p11; 13q22 | Multiple abortions | Castle et al., 1988 ^[52]^ |
| 47 | t(8;13) | 8q21; 13p11 | Azoospermia | Ferguson et al., 2008^[19]^ |
| 48 | t(8;13) | 8q22; 13p11.2 | Azoospermia | Goel et al., 2011 ^[31]^ |
| 49 | t(8;13) | 8q22; 13q22 | Oligozoospermia | Vozdova et al., 2013 ^[23]^ |
| 50 | t(9;13) | 9q31; 13q34 | Recurrent pregnancy loss | [Kochhar](http://www.ncbi.nlm.nih.gov/pubmed/?term=Kochhar%20PK%5BAuthor%5D&cauthor=true&cauthor_uid=22672580) et al., 2013 ^[26]^ |
| 51 | t(9;13) | 9q21.1; 13q21.2 | Spontaneous abortions | Martin et al., 1995 ^[89]^ |
| 52 | t(9;13) | 9p23; 13q21.1 | PGD | Ko et al.,2010 ^[50]^ |
| 53 | t(9;13) | 9q32; 13q32 | Previous miscarriage | Sugiura-Ogasawara et al.,2008^[75]^ |
| 54 | t(9;13) | 9p22; 13q14 | Multiple abortions | Campana et al., 1986 ^[51]^ |
| 55 | t(9;13) | 9q12; 13q13 | Prior miscarriage and PGD | Ikuma et al., 2015 ^[80]^ |
| 56 | t(10;13) | 10p15.1; 13q31.2 | PGD | Ko et al.,2010 ^[50]^ |
| 57 | t(10;13) | 10q24; 13q34 | Previous miscarriage | Sugiura-Ogasawara et al.,2008 ^[75]^ |
| 58 | t(10;13) | 10p13; 13q22 | Fetal losses | Adamoli et al., 1986 ^[53]^ |
| 59 | t(10;13) | 10q23.3; 13q13 | Repeated miscarriage | Goddijn et al., 2004 ^[62]^ |
| 60 | t(10;13) | 10q25; 13q32 | Reproductive disorders | Meza-Espinoza et al., 2008 ^[79]^ |
| 61 | t(10;13) | 10p13; 13p13 | Oligozoospermia | Anton et al., 2008 ^[71]^ |
| 62 | t(11;13) | 11p13; 13q22.3 | Recurrent pregnancy loss | [Kochhar](http://www.ncbi.nlm.nih.gov/pubmed/?term=Kochhar%20PK%5BAuthor%5D&cauthor=true&cauthor_uid=22672580) et al., 2013 ^[26]^ |
| 63 | t(11;13) | 11p13; 13q22.3 | Spontaneous abortion | Stephenson et al., 2006 ^[63]^ |
| 64 | t(11;13) | 11q23; 13q22 | Spontaneous abortion | Davis et al., 1982 ^[61]^ |
| 65 | t(12;13) | 12p13; 13q32 | PGD | [Gianaroli](https://www.ncbi.nlm.nih.gov/pubmed/?term=Gianaroli%20L%5BAuthor%5D&cauthor=true&cauthor_uid=12456624) et al., 2002 ^[70]^ |
| 66 | t(12;13) | 12q23; 13q21 | Repeated miscarriages | Iyer et al., 2007 ^[73]^ |
| 67 | t(13;14) | 13p11; 14q11 | ICSI | Gekas et al., 2001 ^[69]^ |
| 68 | t(13;14) | 13q14.3; 14q32.1 | Miscarriage | Ikuma et al., 2015 ^[80]^ |
| 69 | t(13;14) | 13q22; 14q11.2 | Prior miscarriage and PGD | Ikuma et al., 2015 ^[80]^ |
| 70 | t(13;14) | 13q21;14q21 | PGD | Kyu Lim et al.,2004 ^[46]^ |
| 71 | t(13;15) | 13q14.1; 15q26.3 | Spontaneous abortion | Vozdova et al., 2013 ^[23]^ |
| 72 | t(13;15) | 13q31; 15q23 | Repeated miscarriages | Iyer et al., 2007 ^[73]^ |
| 73 | t(13;15) | 13q21; 15q26 | Spontaneous abortion | Bourrouillou et al., 1986 ^[60]^ |
| 74 | t(13;15) | 13q31; 15q26.2 | Asthenoteratozoospermia | Perrin et al., 2013 ^[57]^ |
| 75 | t(13;15) | 13q32; 15p11.2 | Asthenospermic | Vozdova et al., 2013 ^[23]^ |
| 76 | t(13;15) | 13q14.1; 15q26.1 | Miscarriage | Ikuma et al., 2015 ^[80]^ |
| 77 | t(13;16) | 13q14.1; 16q13 | Recurrent spontaneous pregnancy loss | Gada Saxena et al., 2012 ^[27]^ |
| 78 | t(13;17) | 13q12.3; 17p13.3 | Oligozoospermia | Matsuda et al., 1992 ^[41]^ |
| 79 | t(13;17) | 13p13; 17p12 | Oligozoospermia | Perrin et al., 2013 ^[57]^ |
| 80 | t(13;17) | 13q22; 17q23 | Normal semen | Perrin et al., 2009 ^[38]^ |
| 81 | t(13;17) | 13q14.1; 17q23 | Previous miscarriage | Sugiura-Ogasawara et al.,2008^[75]^ |
| 82 | t(13;22) | 13q22; 22q11 | Reproductive disorders | Meza-Espinoza et al., 2008 ^[79]^ |
| 83 | t(1;14) | 1q44; 14q22 | Oligozoospermia | Vozdova et al., 2013 ^[23]^ |
| 84 | t(1;14) | 1p22; 14q21 | Normal semen | Pellestor et al., 2001 ^[86]^ |
| 85 | t(1;14) | 1q44; 14q11.2 | Severe oligozoospermia | Dul et al., 2012 ^[36]^ |
| 86 | t(2;14) | 2p23.1; 14q31 | ICSI | Gekas et al., 2001 ^[69]^ |
| 87 | t(2;14) | 2p13; 14q11 | Fetal losses | Adamoli et al., 1986 ^[53]^ |
| 88 | t(2;14) | 2p23.1; 14q31 | Infertility | Douet-Guilbert et al., 2005 ^[84]^ |
| 89 | t(2;14) | 2p13; 14q32 | Fetal losses | Adamoli et al., 1986 ^[53]^ |
| 90 | t(2;14) | 2q12; 14q32.33 | Repeated abortions | Manvelyan et al., 2007 ^[34]^ |
| 91 | t(3;14) | 3p12; 14q12 | Recurrent miscarriages | Dutta et al., 2011 ^[32]^ |
| 92 | t(3;14) | 3q21; 14q32 | Infertility or PGD | Brugnon et al., 2006 ^[64]^ |
| 93 | t(3;14) | 3q23; 14q32.2 | PGD | Kyu Lim et al.,2004 ^[46]^ |
| 94 | t(3;14) | 3q25; 14q32 | Normal semen | Godo et al., 2013 ^[9]^ |
| 95 | t(3;14) | 3q27; 14q11 | Oligozoospermia | Matsuda et al., 1992 ^[41]^ |
| 96 | t(4;14) | 4p14; 14q24 | Adverse reproductive outcomes | Gadow et al., 1991 ^[68]^ |
| 97 | t(4;14) | 4q31.3;14q22 | Recurrent fetal wastage | Fryns et al., 1998 ^[25]^ |
| 98 | t(4;14) | 4p14; 14q13 | Recurrent abortion | Portnoï et al., 1988 ^[76]^ |
| 99 | t(4;14) | 4q25; 14q24 | Recurrent spontaneous abortion | Zhang et al., 2015 ^[28]^ |
| 100 | t(5;14) | 5p13; 14q11.2 | PGD | Zhang et al., 2014 ^[39]^ |
| 101 | t(5;14) | 5q32; 14q11.2 | ICSI | Mau et al., 1997 ^[78]^ |
| 102 | t(5;14) | 5p13; 14q23 | Spontaneous abortion | Bourrouillou et al., 1986 ^[60]^ |
| 103 | t(5;14) | 5q11.2; 14q32.1 | Spontaneous abortion | Stephenson et al., 2006 ^[63]^ |
| 104 | t(6;14) | 6p24; 14q22 | A son with t(6;14) | Balkan et al., 1983 ^[59]^ |
| 105 | t(6;14) | 6q13; 14p10 | Oligozoospermia | Li et al., 2012 ^[21]^ |
| 106 | t(6;14) | 6q15; 14q22 | Fetal abnormalities | Zhang et al., 2006 ^[45]^ |
| 107 | t(6;14) | 6q21; 14q13.3 | 2 healthy sons | Olszewska et al., 2017 ^[54]^ |
| 108 | t(6;14) | 6q13; 14q24 | Previous miscarriage | Sugiura-Ogasawara et al.,2008^[75]^ |
| 109 | t(6;14) | 6q24.2;14q24.2 | Missed abortion | Vozdova et al., 2013 ^[23]^ |
| 110 | t(6;14) | 6q27; 14q24.3 | PGD | Zhang et al., 2014 ^[39]^ |
| 111 | t(7;14) | 7q36; 14q11 | Recurrent spontaneous abortions | Tunç et al., 2016 ^[33]^ |
| 112 | t(7;14) | 7qter; 14q22 | Recurrent spontaneous abortions | Tunç et al., 2016 ^[33]^ |
| 113 | t(7;14) | 7q21; 14q13 | Four normal children | Martin et al., 1990 ^[88]^ |
| 114 | t(7;14) | 7q33; 14q32.3 | Recurrent miscarriages | Dutta et al., 2011 ^[32]^ |
| 115 | t(8;14) | 8q22; 14q32 | Normal semen | Godo et al., 2013 ^[9]^ |
| 116 | t(8;14) | 8q22; 14q32 | Not recorded | Anton et al., 2008 ^[71]^ |
| 117 | t(8;14) | 8q21.3; 14q31 | PGD | Kyu Lim et al.,2004 ^[46]^ |
| 118 | t(9;14) | 9q22.1;14q12 | Infertility | Gada Saxena et al., 2012 ^[27]^ |
| 119 | t(9;14) | 9q32; 14p11.2 | Infertility or PGD | Brugnon et al., 2006 ^[64]^ |
| 120 | t(10;14) | 10q24; 14q32 | Normal semen | Perrin et al., 2009 ^[38]^ |
| 121 | t(10;14) | 10q24.2; 14p11.2 | Oligoasthenozoospermia | Godo et al., 2013 ^[9]^ |
| 122 | t(10;14) | 10q26.1;14q13 | Infertility | Gada Saxena et al., 2012 ^[27]^ |
| 123 | t(10;14) | 10q24; 14q32 | Miscarriage | Oliver-Bonet et al., 2004 ^[90]^ |
| 124 | t(10;14) | 10q25; 14q12 | PGD | Zhang et al., 2014 ^[39]^ |
| 125 | t(10;14) | 10q25; 14q32.2 | Recurrent fetal wastage | Fryns et al., 1998 ^[25]^ |
| 126 | t(10;14) | 10q25.2; 14q22 | Miscarriage | Ikuma et al., 2015 ^[80]^ |
| 127 | t(10;14) | 10q24; 14p11.2 | Oligozoospermia | Anton et al., 2008 ^[71]^ |
| 128 | t(12;14) | 12q24.1;14q22.3 | Recurrent fetal wastage | Fryns et al., 1998 ^[25]^ |
| 129 | t(12;14) | 12q13.1; 14q32.1 | Recurrent miscarriage | Pundir et al., 2016 ^[81]^ |
| 130 | t(12;14) | 12q24.1; 14q32 | Miscarriage | Ikuma et al., 2015 ^[80]^ |
| 131 | t(12;14) | 12q22; 14q24.1 | ICSI | Mau et al., 1997 ^[78]^ |
| 132 | t(14;15) | 14q11; 15q32 | Recurrent spontaneous abortions | Tunç et al., 2016 ^[33]^ |
| 133 | t(14;15) | 14q32; 15q13 | Infertility or PGD | Brugnon et al., 2006 ^[64]^ |
| 134 | t(14;15) | 14q24; 15q24 | Recurrent abortions | Soh et al., 1984 ^[43]^ |
| 135 | t(14;18) | 14q32; 14q22.2 | Spontaneous abortion | Schwartz et al., 1983 ^[82]^ |
| 136 | t(14;19) | 14q32; 19q12 | Recurrent spontaneous abortions | Tunç et al., 2016 ^[33]^ |
| 137 | t(14;20) | 14p10; 20q10 | Severe oligozoospermia | Antonelli et al., 2000 ^[58]^ |
| 138 | t(14;20) | 14p11.2; 20q11.2 | PGD | [Gianaroli](https://www.ncbi.nlm.nih.gov/pubmed/?term=Gianaroli%20L%5BAuthor%5D&cauthor=true&cauthor_uid=12456624) et al., 2002 ^[670]^ |
| 139 | t(14;21) | 14q22; 21q22.1 | Infertility | Manvelyan et al., 2007 ^[34]^ |
| 140 | t(1;15) | 1q11; 15p11 | Azoospermia | López-Ginés et al., 1987 ^[35]^ |
| 141 | t(1;15) | 1q21; 15p11.2 | Recurrent miscarriage | Pundir et al., 2016 ^[81]^ |
| 142 | t(1;15) | 1q21.1; 15q26.2 | Spontaneous abortion | Schwartz et al., 1983 ^[82]^ |
| 143 | t(1;15) | 1q43; 15q15 | Extreme OAT | Peschka et al., 1999 ^[83]^ |
| 144 | t(1;15) | 1q21; 15p11 | ICSI | Gekas et al., 2001 ^[69]^ |
| 145 | t(2;15) | 2q21; 15p12 | Recurrent fetal wastage | Fryns et al., 1998 ^[25]^ |
| 146 | t(3;15) | 3p21; 15p11 | PGD | Findikli et al., 2003 ^[67]^ |
| 147 | t(3;15) | 3p25; 15q15 | Infertility | Blanco et al., 2000 ^[85]^ |
| 148 | t(3;15) | 3p22; 15q26.2 | Previous miscarriage | Sugiura-Ogasawara et al.,2008^[75]^ |
| 149 | t(3;15) | 3q24; 15q25 | PGD | Ko et al.,2010 ^[50]^ |
| 150 | t(3;15) | 3q24; 15q21 | Reproductive disorders | Meza-Espinoza et al., 2008 ^[79]^ |
| 151 | t(3;15) | 3q26.2; 15q26.1 | Inherited from his father | Estop et al., 1995 ^[66]^ |
| 152 | t(4;15) | 4p16; 15q22.2 | Oligoasthenoteratozoospermia | Dohle et al., 2002 ^[65]^ |
| 153 | t(4;15) | 4q21; 15q15 | PGD | [Gianaroli](https://www.ncbi.nlm.nih.gov/pubmed/?term=Gianaroli%20L%5BAuthor%5D&cauthor=true&cauthor_uid=12456624) et al., 2002 ^[70]^ |
| 154 | t(4;15) | 4p15; 15q24 | Spontaneous abortion | Bourrouillou et al., 1986 ^[60]^ |
| 155 | t(4;15) | 4q25;15q26.3 | Recurrent pregnancy loss | [Kochhar](http://www.ncbi.nlm.nih.gov/pubmed/?term=Kochhar%20PK%5BAuthor%5D&cauthor=true&cauthor_uid=22672580) et al., 2013 ^[26]^ |
| 156 | t(4;15) | 4q31.3; 15q26.1 | Recurrent miscarriage | Pundir et al., 2016 ^[81]^ |
| 157 | t(4;15) | 4q12; 15q23 | Recurrent miscarriage | Pundir et al., 2016 ^[81]^ |
| 158 | t(4;15) | 4p15.3; 15q25 | Miscarriage | Ikuma et al., 2015 ^[80]^ |
| 159 | t(5;15) | 5p13.3; 15q15.3 | PGD | Ko et al.,2010 ^[50]^ |
| 160 | t(5;15) | 5p15.3; 15q21.1 | PGD | Ko et al.,2010 ^[50]^ |
| 161 | t(5;15) | 5q35; 15q22 | PGD | Escudero et al.,2003 ^[37]^ |
| 162 | t(5;15) | 5q35; 15q26.2 | Normospermic | Vozdova et al., 2013 ^[23]^ |
| 163 | t(6;15) | 6p22; 15q26.3 | Normal semen | Vegetti et al., 2000 ^[44]^ |
| 164 | t(6;15) | 6q25; 15q14 | Recurrent spontaneous pregnancy loss | Gada Saxena et al., 2012 ^[27]^ |
| 165 | t(6;15) | 6q23; 15q21.1 | Miscarriage | Ikuma et al., 2015 ^[80]^ |
| 166 | t(6;15) | 6p21; 15q26.1 | Recurrent fetal wastage | Fryns et al., 1998 ^[25]^ |
| 167 | t(7;15) | 7p15; 15q15 | Oligozoospermia | Li et al., 2012 ^[21]^ |
| 168 | t(7;15) | 7p15.1; 15q13 | Oligoasthenoteratospermia | Perrin et al., 2013 ^[57]^ |
| 169 | t(7;15) | 7p15.3; 15q26 | Infertility or PGD | Brugnon et al., 2006 ^[64]^ |
| 170 | t(7;15) | 7q32; 15q24 | 2 early miscarriages | Olszewska et al., 2017 ^[54]^ |
| 171 | t(8;15) | 8p23.1; 15q24 | Recurrent miscarriage | Pundir et al., 2016 ^[81]^ |
| 172 | t(8;15) | 8p22; 15q21 | Two spontaneous abortions | Brandriff et al., 1986 ^[56]^ |
| 173 | t(9;15) | 9p14; 15q22 | Recurrent spontaneous abortion | Zhang et al., 2015 ^[28]^ |
| 174 | t(9;15) | 9q32; 15q24 | Multiple abortions | Castle et al., 1988 ^[52]^ |
| 175 | t(10;15) | 10p15; 15q22 | Spontaneous abortion | Bourrouillou et al., 1986 ^[60]^ |
| 176 | t(10;15) | 10p14; 15q26 | Normal semen | Perrin et al., 2009 ^[38]^ |
| 177 | t(10;15) | 10q26;15q12 | Infertility | Baccetti et al., 2003 ^[20]^ |
| 178 | t(10;15) | 10q25.2; 15q12 | Spontaneous abortion | Vozdova et al., 2013 ^[23]^ |
| 179 | t(10;15) | 10q26;15q15 | Infertility | Gada Saxena et al., 2012 ^[27]^ |
| 180 | t(11;15) | 11p15; 15p13 | Infertility | Manvelyan et al., 2007 ^[34]^ |
| 181 | t(11;15) | 11q14.2; 15q25 | Multiple abortions | Castle et al., 1988 ^[52]^ |
| 182 | t(12;15) | 12p13; 15p11.1 | PGD | Findikli et al., 2003 ^[67]^ |
| 183 | t(12;15) | 12q12; 15q26.1 | PGD | Ko et al.,2010 ^[50]^ |
| 184 | t(12;15) | 12q13; 15q26 | PGD | Kyu Lim et al., 2004 ^[46]^ |
| 185 | t(12;15) | 12p13.3; 15q24 | Infertility | Manvelyan et al., 2007 ^[34]^ |
| 186 | t(12;15) | 12q24.31;15q11.2 | Miscarriage | Ikuma et al., 2015 ^[80]^ |
| 187 | t(15;16) | 15p10; 16q10 | Asthenozoospermia | Perrin et al., 2013 ^[57]^ |
| 188 | t(15;16) | 15q26.3; 16q13 | Severe oligozoospermia | Matsuda et al., 1992 ^[41]^ |
| 189 | t(15;17) | 15p11.2; 17q21.3 | Oligozoospermia | Kuroda et al., 2014 ^[42]^ |
| 190 | t(15;17) | 15q11; 17p12 | PGD | Yakut et al., 2006 ^[49]^ |
| 191 | t(15;17) | 15q21; 17p12 | PGD | Kyu Lim et al.,2004 ^[46]^ |
| 192 | t(15;17) | 15q21; 17q25 | Miscarriage | Cora et al., 2002 ^[91]^ |
| 193 | t(15;17) | 15q26.3; 17q23.1 | 2 early miscarriages | Olszewska et al., 2017 ^[54]^ |
| 194 | t(15;19) | 15q11; 19q11 | Oligoasthenoteratozoospermia | Machev et al., 2005 ^[77]^ |
| 195 | t(15;20) | 15q11.2; 20q11.2 | Multiply handicapped baby | Goldman et al., 1993 ^[92]^ |
| 196 | t(15;20) | 15q22; 20q13 | Recurrent pregnancy loss | Sider et al., 1988 ^[47]^ |
| 197 | t(15;21) | 15q24; 21q22.3 | Oligozoospermia | Dul et al., 2012 ^[36]^ |
| 198 | t(15;22) | 15q13; 22q13.3 | Recurrent miscarriage | Pundir et al., 2016 ^[81]^ |
| 199 | t(15;22) | 15q22; 22q13 | PGD | Escudero et al.,2003 ^[37]^ |
| 200 | t(15;22) | 15q26.1; 22q11.2 | Early pregnancy loss | Martin et al., 1993 ^[74]^ |
| 201 | t(X;15) | Xq28; 15q22 | Adverse reproductive outcomes | Gadow et al., 1991 ^[68]^ |

ICSI= Intracytoplasmic sperm injection, OAT=Oligoasthenoteratospermia, PGD= Preimplantation genetic diagnosis

**References**

[24] [Mierla D](https://www.ncbi.nlm.nih.gov/pubmed/?term=Mierla%20D%5BAuthor%5D&cauthor=true&cauthor_uid=24696767), [Jardan D](https://www.ncbi.nlm.nih.gov/pubmed/?term=Jardan%20D%5BAuthor%5D&cauthor=true&cauthor_uid=24696767), [Stoian V](https://www.ncbi.nlm.nih.gov/pubmed/?term=Stoian%20V%5BAuthor%5D&cauthor=true&cauthor_uid=24696767). Chromosomal abnormality in men with impaired spermatogenesis. [Int J Fertil Steril](https://www.ncbi.nlm.nih.gov/pubmed/24696767) 2014; 8:35-42.

[25] Fryns JP, Van Buggenhout G. [Structural chromosome rearrangements in couples with recurrent fetal wastage.](https://www.ncbi.nlm.nih.gov/pubmed/9989862) Eur J Obstet Gynecol Reprod Biol 1998; 81:171-6.

[26] [Kochhar PK](http://www.ncbi.nlm.nih.gov/pubmed/?term=Kochhar%20PK%5BAuthor%5D&cauthor=true&cauthor_uid=22672580), [Ghosh P](http://www.ncbi.nlm.nih.gov/pubmed/?term=Ghosh%20P%5BAuthor%5D&cauthor=true&cauthor_uid=22672580). Reproductive outcome of couples with recurrent miscarriage and balanced chromosomal abnormalities. [J Obstet Gynaecol Res](http://www.ncbi.nlm.nih.gov/pubmed/22672580) 2013; 39:113-20.

[27] [Gada Saxena S](https://www.ncbi.nlm.nih.gov/pubmed/?term=Gada%20Saxena%20S%5BAuthor%5D&cauthor=true&cauthor_uid=22695313), [Desai K](https://www.ncbi.nlm.nih.gov/pubmed/?term=Desai%20K%5BAuthor%5D&cauthor=true&cauthor_uid=22695313), [Shewale L](https://www.ncbi.nlm.nih.gov/pubmed/?term=Shewale%20L%5BAuthor%5D&cauthor=true&cauthor_uid=22695313), et al. Chromosomal aberrations in 2000 couples of Indian ethnicity with reproductive failure. [Reprod Biomed Online](https://www.ncbi.nlm.nih.gov/pubmed/?term=Chromosomal+aberrations+in+2000+couples+of+Indian+ethnicity+with+reproductive+failure) 2012; 25:209-18.

[28] Zhang M, [Fan HT](https://www.ncbi.nlm.nih.gov/pubmed/?term=Fan%20HT%5BAuthor%5D&cauthor=true&cauthor_uid=26662410), [Zhang QS](https://www.ncbi.nlm.nih.gov/pubmed/?term=Zhang%20QS%5BAuthor%5D&cauthor=true&cauthor_uid=26662410), [et](https://www.ncbi.nlm.nih.gov/pubmed/?term=Li%20RW%5BAuthor%5D&cauthor=true&cauthor_uid=26662410) al. Genetic screening and evaluation for chromosomal abnormalities of infertile males in Jilin Province, China. Genet Mol Res 2015; 14: 16178-84.

[29] [Niroumanesh S](https://www.ncbi.nlm.nih.gov/pubmed/?term=Niroumanesh%20S%5BAuthor%5D&cauthor=true&cauthor_uid=21245604), [Mehdipour P](https://www.ncbi.nlm.nih.gov/pubmed/?term=Mehdipour%20P%5BAuthor%5D&cauthor=true&cauthor_uid=21245604), [Farajpour A](https://www.ncbi.nlm.nih.gov/pubmed/?term=Farajpour%20A%5BAuthor%5D&cauthor=true&cauthor_uid=21245604), et al. A cytogenetic study of couples with repeated spontaneous abortions. [Ann Saudi Med](https://www.ncbi.nlm.nih.gov/pubmed/21245604) 2011; 31:77-9.

[30] [Poli MN](http://www.ncbi.nlm.nih.gov/pubmed/?term=Poli%20MN%5BAuthor%5D&cauthor=true&cauthor_uid=27244763), [Miranda LA](https://www.ncbi.nlm.nih.gov/pubmed/?term=Miranda%20LA%5BAuthor%5D&cauthor=true&cauthor_uid=27244763), [Gil ED](https://www.ncbi.nlm.nih.gov/pubmed/?term=Gil%20ED%5BAuthor%5D&cauthor=true&cauthor_uid=27244763), et al. Male cytogenetic evaluation prior to assisted reproduction procedures performed in Mar del Plata, Argentina. [JBRA Assist Reprod](http://www.ncbi.nlm.nih.gov/pubmed/27244763) 2016; 20:62-5.

[31] [Goel H](https://www.ncbi.nlm.nih.gov/pubmed/?term=Goel%20H%5BAuthor%5D&cauthor=true&cauthor_uid=21219388), [Phadke SR](https://www.ncbi.nlm.nih.gov/pubmed/?term=Phadke%20SR%5BAuthor%5D&cauthor=true&cauthor_uid=21219388). Reciprocal balanced translocation: infertility and recurrent spontaneous abortions in a family. [Andrologia](https://www.ncbi.nlm.nih.gov/pubmed/?term=Reciprocal+balanced+translocation%3A+infertility+and+recurrent+spontaneous+abortions+in+a+family) 2011;43:75-7.

[32] [Dutta UR](https://www.ncbi.nlm.nih.gov/pubmed/?term=Dutta%20UR%5BAuthor%5D&cauthor=true&cauthor_uid=20931274), [Rajitha P](https://www.ncbi.nlm.nih.gov/pubmed/?term=Rajitha%20P%5BAuthor%5D&cauthor=true&cauthor_uid=20931274), [Pidugu VK](https://www.ncbi.nlm.nih.gov/pubmed/?term=Pidugu%20VK%5BAuthor%5D&cauthor=true&cauthor_uid=20931274), [et](https://www.ncbi.nlm.nih.gov/pubmed/?term=Dalal%20AB%5BAuthor%5D&cauthor=true&cauthor_uid=20931274) al. Cytogenetic abnormalities in 1162 couples with recurrent miscarriages in southern region of India: report and review. [J Assist Reprod Genet](https://www.ncbi.nlm.nih.gov/pubmed/?term=Cytogenetic+abnormalities+in+1162+couples+with+recurrent+miscarriages+in+Southern+region+of+India%3A+report+and+review) 2011; 28:145-9.

[33] [Tunç E](https://www.ncbi.nlm.nih.gov/pubmed/?term=Tun%C3%A7%20E%5BAuthor%5D&cauthor=true&cauthor_uid=26874988), [Tanrıverdi N](https://www.ncbi.nlm.nih.gov/pubmed/?term=Tanr%C4%B1verdi%20N%5BAuthor%5D&cauthor=true&cauthor_uid=26874988), [Demirhan O](https://www.ncbi.nlm.nih.gov/pubmed/?term=Demirhan%20O%5BAuthor%5D&cauthor=true&cauthor_uid=26874988), et al. Chromosomal analyses of 1510 couples who have experienced recurrent spontaneous abortions. [Reprod Biomed Online](https://www.ncbi.nlm.nih.gov/pubmed/?term=Chromosomal+analyses+of+1510+couples+who+have+experienced+recurrent+spontaneous+abortions) 2016; 32:414-9.

[34] [Manvelyan M](https://www.ncbi.nlm.nih.gov/pubmed/?term=Manvelyan%20M%5BAuthor%5D&cauthor=true&cauthor_uid=17487417), [Schreyer I](https://www.ncbi.nlm.nih.gov/pubmed/?term=Schreyer%20I%5BAuthor%5D&cauthor=true&cauthor_uid=17487417), [Höls-Herpertz I](https://www.ncbi.nlm.nih.gov/pubmed/?term=H%C3%B6ls-Herpertz%20I%5BAuthor%5D&cauthor=true&cauthor_uid=17487417), et al. Forty-eight new cases with infertility due to balanced chromosomal rearrangements: detailed molecular cytogenetic analysis of the 90 involved breakpoints. [Int J Mol Med](https://www.ncbi.nlm.nih.gov/pubmed/?term=Forty-eight+new+cases+with+infertility+due+to+balanced+chromosomal+rearrangements%3A+Detailed+molecular+cytogenetic+analysis+of+the+90+involved+breakpoints) 2007; 19:855-64.

[35] [López-Ginés C](https://www.ncbi.nlm.nih.gov/pubmed/?term=L%C3%B3pez-Gin%C3%A9s%20C%5BAuthor%5D&cauthor=true&cauthor_uid=3679215), [Gil R](https://www.ncbi.nlm.nih.gov/pubmed/?term=Gil%20R%5BAuthor%5D&cauthor=true&cauthor_uid=3679215), [Gregori-Romero M](https://www.ncbi.nlm.nih.gov/pubmed/?term=Gregori-Romero%20M%5BAuthor%5D&cauthor=true&cauthor_uid=3679215), [et](https://www.ncbi.nlm.nih.gov/pubmed/?term=Pellin%20A%5BAuthor%5D&cauthor=true&cauthor_uid=3679215) al. An azoospermic male with reciprocal translocation t(1;15) (q11;p11). [Hum Genet](https://www.ncbi.nlm.nih.gov/pubmed/3679215) 1987;77:294.

[36] Dul EC, van Echten-Arends J, Groen H, et al. Chromosomal abnormalities in azoospermic and non-azoospermic infertile men: numbers needed to be screened to prevent adverse pregnancy outcomes. [Hum Reprod](https://www.ncbi.nlm.nih.gov/pubmed/?term=Chromosomal+abnormalities+in+azoospermic+and+non-azoospermic+infertile+men%3A+numbers+needed+to+be+screened+to+prevent+adverse+pregnancy+outcomes) 2012; 27:2850-6.

[37] [Escudero T](https://www.ncbi.nlm.nih.gov/pubmed/?term=Escudero%20T%5BAuthor%5D&cauthor=true&cauthor_uid=12801555), [Abdelhadi I](https://www.ncbi.nlm.nih.gov/pubmed/?term=Abdelhadi%20I%5BAuthor%5D&cauthor=true&cauthor_uid=12801555), [Sandalinas M](https://www.ncbi.nlm.nih.gov/pubmed/?term=Sandalinas%20M%5BAuthor%5D&cauthor=true&cauthor_uid=12801555), [et](https://www.ncbi.nlm.nih.gov/pubmed/?term=Munn%C3%A9%20S%5BAuthor%5D&cauthor=true&cauthor_uid=12801555) al. Predictive value of sperm fluorescence in situ hybridization analysis on the outcome of preimplantation genetic diagnosis for translocations. [Fertil Steril](https://www.ncbi.nlm.nih.gov/pubmed/12801555) 2003; 79 Suppl 3:1528-34.

[38] [Perrin A](https://www.ncbi.nlm.nih.gov/pubmed/?term=Perrin%20A%5BAuthor%5D&cauthor=true&cauthor_uid=18706548), [Caer E](https://www.ncbi.nlm.nih.gov/pubmed/?term=Caer%20E%5BAuthor%5D&cauthor=true&cauthor_uid=18706548), [Oliver-Bonet M](https://www.ncbi.nlm.nih.gov/pubmed/?term=Oliver-Bonet%20M%5BAuthor%5D&cauthor=true&cauthor_uid=18706548), et al. DNA fragmentation and meiotic segregation in sperm of carriers of a chromosomal structural abnormality. [Fertil Steril](https://www.ncbi.nlm.nih.gov/pubmed/18706548) 2009; 92:583-9.

[39] [Zhang Y](https://www.ncbi.nlm.nih.gov/pubmed/?term=Zhang%20Y%5BAuthor%5D&cauthor=true&cauthor_uid=25131559), [Zhu S](https://www.ncbi.nlm.nih.gov/pubmed/?term=Zhu%20S%5BAuthor%5D&cauthor=true&cauthor_uid=25131559), [Wu J](https://www.ncbi.nlm.nih.gov/pubmed/?term=Wu%20J%5BAuthor%5D&cauthor=true&cauthor_uid=25131559), et al. Quadrivalent asymmetry in reciprocal translocation carriers predicts meiotic segregation patterns in cleavage stage embryos. [Reprod Biomed Online](https://www.ncbi.nlm.nih.gov/pubmed/?term=Quadrivalent+asymmetry+in+reciprocal+translocation+carriers+predicts+meiotic+segregation+patterns+in+cleavage+stage+embryos) 2014;29:490-8.

[40] [Haapaniemi Kouru K](https://www.ncbi.nlm.nih.gov/pubmed/?term=Haapaniemi%20Kouru%20K%5BAuthor%5D&cauthor=true&cauthor_uid=28336162), [Malmgren H](https://www.ncbi.nlm.nih.gov/pubmed/?term=Malmgren%20H%5BAuthor%5D&cauthor=true&cauthor_uid=28336162), [White I](https://www.ncbi.nlm.nih.gov/pubmed/?term=White%20I%5BAuthor%5D&cauthor=true&cauthor_uid=28336162), et al. Meiotic segregation analyses of reciprocal translocations in spermatozoa and embryos: no support for predictive value regarding PGD outcome. [Reprod Biomed Online](https://www.ncbi.nlm.nih.gov/pubmed/?term=Meiotic+segregation+analyses+of+reciprocal+translocations+in+spermatozoa+and+embryos%3A+no+support+for+predictive+value+regarding+PGD+outcome) 2017;34:645-52.

[41] [Matsuda T](https://www.ncbi.nlm.nih.gov/pubmed/?term=Matsuda%20T%5BAuthor%5D&cauthor=true&cauthor_uid=1524005), [Horii Y](https://www.ncbi.nlm.nih.gov/pubmed/?term=Horii%20Y%5BAuthor%5D&cauthor=true&cauthor_uid=1524005), [Ogura K](https://www.ncbi.nlm.nih.gov/pubmed/?term=Ogura%20K%5BAuthor%5D&cauthor=true&cauthor_uid=1524005), et al. Chromosomal survey of 1001 subfertile males: incidence and clinical features of males with chromosomal anomalies. [Hinyokika Kiyo](https://www.ncbi.nlm.nih.gov/pubmed/1524005) 1992;38:803-9.

[42] [Kuroda S](https://www.ncbi.nlm.nih.gov/pubmed/?term=Kuroda%20S%5BAuthor%5D&cauthor=true&cauthor_uid=25142955), [Yumura Y](https://www.ncbi.nlm.nih.gov/pubmed/?term=Yumura%20Y%5BAuthor%5D&cauthor=true&cauthor_uid=25142955), [Yasuda K](https://www.ncbi.nlm.nih.gov/pubmed/?term=Yasuda%20K%5BAuthor%5D&cauthor=true&cauthor_uid=25142955), et al. Clinical investigation of male infertile patients with chromosomal anomalies. [Hinyokika Kiyo](https://www.ncbi.nlm.nih.gov/pubmed/25142955) 2014;60:309-13.

[43] [Soh K](https://www.ncbi.nlm.nih.gov/pubmed/?term=Soh%20K%5BAuthor%5D&cauthor=true&cauthor_uid=6515649), [Yajima A](https://www.ncbi.nlm.nih.gov/pubmed/?term=Yajima%20A%5BAuthor%5D&cauthor=true&cauthor_uid=6515649), [Ozawa N](https://www.ncbi.nlm.nih.gov/pubmed/?term=Ozawa%20N%5BAuthor%5D&cauthor=true&cauthor_uid=6515649), et al. Chromosome analysis in couples with recurrent abortions. [Tohoku J Exp Med](https://www.ncbi.nlm.nih.gov/pubmed/6515649) 1984;144:151-63.

[44] [Vegetti W](https://www.ncbi.nlm.nih.gov/pubmed/?term=Vegetti%20W%5BAuthor%5D&cauthor=true&cauthor_uid=10655307), [Van Assche E](https://www.ncbi.nlm.nih.gov/pubmed/?term=Van%20Assche%20E%5BAuthor%5D&cauthor=true&cauthor_uid=10655307), [Frias A](https://www.ncbi.nlm.nih.gov/pubmed/?term=Frias%20A%5BAuthor%5D&cauthor=true&cauthor_uid=10655307), et al. Correlation between semen parameters and sperm aneuploidy rates investigated by fluorescence in-situ hybridization in infertile men. [Hum Reprod](https://www.ncbi.nlm.nih.gov/pubmed/?term=Correlation+between+semen+parameters+and+sperm+aneuploidy+rates+investigated+by+fluorescence+in-situ+hybridization+in+infertile+men) 2000;15:351-65.

[45] [Zhang YP](https://www.ncbi.nlm.nih.gov/pubmed/?term=Zhang%20YP%5BAuthor%5D&cauthor=true&cauthor_uid=17181967), [Xu JZ](https://www.ncbi.nlm.nih.gov/pubmed/?term=Xu%20JZ%5BAuthor%5D&cauthor=true&cauthor_uid=17181967), [Yin M](https://www.ncbi.nlm.nih.gov/pubmed/?term=Yin%20M%5BAuthor%5D&cauthor=true&cauthor_uid=17181967), et al. Pregnancy outcomes of 194 couples with balanced translocations. [Zhonghua Fu Chan Ke Za Zhi](https://www.ncbi.nlm.nih.gov/pubmed/17181967) 2006;41:592-6.

[46] [Kyu Lim C](https://www.ncbi.nlm.nih.gov/pubmed/?term=Kyu%20Lim%20C%5BAuthor%5D&cauthor=true&cauthor_uid=15300749), [Hyun Jun J](https://www.ncbi.nlm.nih.gov/pubmed/?term=Hyun%20Jun%20J%5BAuthor%5D&cauthor=true&cauthor_uid=15300749), [Mi Min D](https://www.ncbi.nlm.nih.gov/pubmed/?term=Mi%20Min%20D%5BAuthor%5D&cauthor=true&cauthor_uid=15300749), [et](https://www.ncbi.nlm.nih.gov/pubmed/?term=Kang%20IS%5BAuthor%5D&cauthor=true&cauthor_uid=15300749) al. Efficacy and clinical outcome of preimplantation genetic diagnosis using FISH for couples of reciprocal and Robertsonian translocations: the Korean experience. [Prenat Diagn](https://www.ncbi.nlm.nih.gov/pubmed/?term=Efficacy+and+clinical+outcome+of+preimplantation+genetic+diagnosis+using+FISH+for+couples+of+reciprocal+and+Robertsonian+translocations%3A+the+Korean+experience) 2004;24:556-61.

[47] [Sider D](https://www.ncbi.nlm.nih.gov/pubmed/?term=Sider%20D%5BAuthor%5D&cauthor=true&cauthor_uid=3201300), [Wilson WG](https://www.ncbi.nlm.nih.gov/pubmed/?term=Wilson%20WG%5BAuthor%5D&cauthor=true&cauthor_uid=3201300), [Sudduth K](https://www.ncbi.nlm.nih.gov/pubmed/?term=Sudduth%20K%5BAuthor%5D&cauthor=true&cauthor_uid=3201300), et al. Cytogenetic studies in couples with recurrent pregnancy loss. [South Med J](https://www.ncbi.nlm.nih.gov/pubmed/?term=Sider+D%2C+Wilson+WG) 1988;81:1521-4.

[48] [Midro AT](https://www.ncbi.nlm.nih.gov/pubmed/?term=Midro%20AT%5BAuthor%5D&cauthor=true&cauthor_uid=16411217), [Wiland E](https://www.ncbi.nlm.nih.gov/pubmed/?term=Wiland%20E%5BAuthor%5D&cauthor=true&cauthor_uid=16411217), [Panasiuk B](https://www.ncbi.nlm.nih.gov/pubmed/?term=Panasiuk%20B%5BAuthor%5D&cauthor=true&cauthor_uid=16411217), et al. Risk evaluation of carriers with chromosome reciprocal translocation t(7;13)(q34;q13) and concomitant meiotic segregation analyzed by FISH on ejaculated spermatozoa. [Am J Med Genet A](https://www.ncbi.nlm.nih.gov/pubmed/?term=Risk+Evaluation+of+Carriers+With+Chromosome+Reciprocal+Translocation+t(7%3B13)(q34%3Bq13)+and+Concomitant+Meiotic+Segregation+Analyzed+by+FISH+on+Ejaculated+Spermatozoa) 2006;140:245-56.

[49] [Yakut T](https://www.ncbi.nlm.nih.gov/pubmed/?term=Yakut%20T%5BAuthor%5D&cauthor=true&cauthor_uid=16596678), [Ercelen N](https://www.ncbi.nlm.nih.gov/pubmed/?term=Ercelen%20N%5BAuthor%5D&cauthor=true&cauthor_uid=16596678), [Acar H](https://www.ncbi.nlm.nih.gov/pubmed/?term=Acar%20H%5BAuthor%5D&cauthor=true&cauthor_uid=16596678), et al. Meiotic segregation analysis of reciprocal translocations both in sperms and blastomeres. [Am J Med Genet A](https://www.ncbi.nlm.nih.gov/pubmed/16596678) 2006;140:1074-82.

[50] [Ko DS](https://www.ncbi.nlm.nih.gov/pubmed/?term=Ko%20DS%5BAuthor%5D&cauthor=true&cauthor_uid=20503317), [Cho JW](https://www.ncbi.nlm.nih.gov/pubmed/?term=Cho%20JW%5BAuthor%5D&cauthor=true&cauthor_uid=20503317), [Park SY](https://www.ncbi.nlm.nih.gov/pubmed/?term=Park%20SY%5BAuthor%5D&cauthor=true&cauthor_uid=20503317), et al. Clinical outcomes of preimplantation genetic diagnosis (PGD) and analysis of meiotic segregation modes in reciprocal translocation carriers. [Am J Med Genet A](https://www.ncbi.nlm.nih.gov/pubmed/?term=Clinical+Outcomes+of+Preimplantation+Genetic+Diagnosis+(PGD)+and+Analysis+of+Meiotic+Segregation+Modes+in+Reciprocal+Translocation+Carriers) 2010;152A:1428-33.

[51] [Campana M](https://www.ncbi.nlm.nih.gov/pubmed/?term=Campana%20M%5BAuthor%5D&cauthor=true&cauthor_uid=3717213), [Serra A](https://www.ncbi.nlm.nih.gov/pubmed/?term=Serra%20A%5BAuthor%5D&cauthor=true&cauthor_uid=3717213), [Neri G](https://www.ncbi.nlm.nih.gov/pubmed/?term=Neri%20G%5BAuthor%5D&cauthor=true&cauthor_uid=3717213). Role of chromosome aberrations in recurrent abortion: a study of 269 balanced translocations. [Am J Med Genet](https://www.ncbi.nlm.nih.gov/pubmed/?term=Role+of+Chromosome+Aberrations+in+Recurrent+Abortion%3A+A+Study+of+269+Balanced+Translocations) 1986;24:341-56.

[52] [Castle D](https://www.ncbi.nlm.nih.gov/pubmed/?term=Castle%20D%5BAuthor%5D&cauthor=true&cauthor_uid=3376998), [Bernstein R](https://www.ncbi.nlm.nih.gov/pubmed/?term=Bernstein%20R%5BAuthor%5D&cauthor=true&cauthor_uid=3376998). Cytogenetic analysis of 688 couples experiencing multiple spontaneous abortions. [Am J Med Genet](https://www.ncbi.nlm.nih.gov/pubmed/?term=Cytogenetic+Analysis+of+688+Couples+Experiencing+Multiple+Spontaneous+Abortions) 1988;29:549-56.

[53] [Adamoli A](https://www.ncbi.nlm.nih.gov/pubmed/?term=Adamoli%20A%5BAuthor%5D&cauthor=true&cauthor_uid=3558760), [Bernardi F](https://www.ncbi.nlm.nih.gov/pubmed/?term=Bernardi%20F%5BAuthor%5D&cauthor=true&cauthor_uid=3558760), [Chiaffoni G](https://www.ncbi.nlm.nih.gov/pubmed/?term=Chiaffoni%20G%5BAuthor%5D&cauthor=true&cauthor_uid=3558760), et al. Reproductive failure and parental chromosome abnormalities. [Hum Reprod](https://www.ncbi.nlm.nih.gov/pubmed/3558760) 1986;1:99-102.

[54] [Olszewska M](https://www.ncbi.nlm.nih.gov/pubmed/?term=Olszewska%20M%5BAuthor%5D&cauthor=true&cauthor_uid=26908061), [Barciszewska MZ](https://www.ncbi.nlm.nih.gov/pubmed/?term=Barciszewska%20MZ%5BAuthor%5D&cauthor=true&cauthor_uid=26908061), [Fraczek M](https://www.ncbi.nlm.nih.gov/pubmed/?term=Fraczek%20M%5BAuthor%5D&cauthor=true&cauthor_uid=26908061), et al. Global methylation status of sperm DNA in carriers of chromosome structural aberrations. [Asian J Androl](https://www.ncbi.nlm.nih.gov/pubmed/?term=Global+methylation+status+of+sperm+DNA+in+carriers+of+chromosome+structural+aberrations) 2017;19:117-24.

[55] [Pellestor F](https://www.ncbi.nlm.nih.gov/pubmed/?term=Pellestor%20F%5BAuthor%5D&cauthor=true&cauthor_uid=2818710), [Sèle B](https://www.ncbi.nlm.nih.gov/pubmed/?term=S%C3%A8le%20B%5BAuthor%5D&cauthor=true&cauthor_uid=2818710), [Jalbert H](https://www.ncbi.nlm.nih.gov/pubmed/?term=Jalbert%20H%5BAuthor%5D&cauthor=true&cauthor_uid=2818710), et al. Direct segregation analysis of reciprocal translocations: a study of 283 sperm karyotypes from four carriers. [Am J Hum Genet](https://www.ncbi.nlm.nih.gov/pubmed/?term=Direct+Segregation+Analysis+of+Reciprocal+Translocations%3A+A+Study+of+283+Sperm+Karyotypes+from+Four+Carriers) 1989;44:464-73.

[56] [Brandriff B](https://www.ncbi.nlm.nih.gov/pubmed/?term=Brandriff%20B%5BAuthor%5D&cauthor=true&cauthor_uid=3946422), [Gordon L](https://www.ncbi.nlm.nih.gov/pubmed/?term=Gordon%20L%5BAuthor%5D&cauthor=true&cauthor_uid=3946422), [Ashworth LK](https://www.ncbi.nlm.nih.gov/pubmed/?term=Ashworth%20LK%5BAuthor%5D&cauthor=true&cauthor_uid=3946422), et al. Cytogenetics of human sperm: meiotic segregation in two translocation carriers. [Am J Hum Genet](https://www.ncbi.nlm.nih.gov/pubmed/3946422) 1986;38:197-208.

[57] [Perrin A](https://www.ncbi.nlm.nih.gov/pubmed/?term=Perrin%20A%5BAuthor%5D&cauthor=true&cauthor_uid=23785022), [Nguyen MH](https://www.ncbi.nlm.nih.gov/pubmed/?term=Nguyen%20MH%5BAuthor%5D&cauthor=true&cauthor_uid=23785022), [Bujan L](https://www.ncbi.nlm.nih.gov/pubmed/?term=Bujan%20L%5BAuthor%5D&cauthor=true&cauthor_uid=23785022), et al. DNA fragmentation is higher in spermatozoa with chromosomally unbalanced content in men with a structural chromosomal rearrangement. [Andrology](https://www.ncbi.nlm.nih.gov/pubmed/?term=DNA+fragmentation+is+higher+in+spermatozoa+with+chromosomally+unbalanced+content+in+men+with+a+structural+chromosomal+rearrangement) 2013;1:632-8.

[58] [Antonelli A](https://www.ncbi.nlm.nih.gov/pubmed/?term=Antonelli%20A%5BAuthor%5D&cauthor=true&cauthor_uid=11097433), [Gandini L](https://www.ncbi.nlm.nih.gov/pubmed/?term=Gandini%20L%5BAuthor%5D&cauthor=true&cauthor_uid=11097433), [Petrinelli P](https://www.ncbi.nlm.nih.gov/pubmed/?term=Petrinelli%20P%5BAuthor%5D&cauthor=true&cauthor_uid=11097433), et al. Chromosomal alterations and male infertility. [J Endocrinol Invest](https://www.ncbi.nlm.nih.gov/pubmed/11097433) 2000;23:677-83.

[59] [Balkan W](https://www.ncbi.nlm.nih.gov/pubmed/?term=Balkan%20W%5BAuthor%5D&cauthor=true&cauthor_uid=6862439), [Martin RH](https://www.ncbi.nlm.nih.gov/pubmed/?term=Martin%20RH%5BAuthor%5D&cauthor=true&cauthor_uid=6862439). Chromosome segregation into the spermatozoa of two men heterozygous for different reciprocal translocations. [Hum Genet](https://www.ncbi.nlm.nih.gov/pubmed/6862439) 1983;63:345-8.

[60] [Bourrouillou G](https://www.ncbi.nlm.nih.gov/pubmed/?term=Bourrouillou%20G%5BAuthor%5D&cauthor=true&cauthor_uid=3793103), [Colombies P](https://www.ncbi.nlm.nih.gov/pubmed/?term=Colombies%20P%5BAuthor%5D&cauthor=true&cauthor_uid=3793103), [Dastugue N](https://www.ncbi.nlm.nih.gov/pubmed/?term=Dastugue%20N%5BAuthor%5D&cauthor=true&cauthor_uid=3793103). Chromosome studies in 2136 couples with spontaneous abortions. [Hum Genet](https://www.ncbi.nlm.nih.gov/pubmed/?term=Chromosome+studies+in+2136+couples+with+spontaneous+abortions) 1986;74:399-401.

[61] [Davis JR](https://www.ncbi.nlm.nih.gov/pubmed/?term=Davis%20JR%5BAuthor%5D&cauthor=true&cauthor_uid=7051833), [Weinstein L](https://www.ncbi.nlm.nih.gov/pubmed/?term=Weinstein%20L%5BAuthor%5D&cauthor=true&cauthor_uid=7051833), [Veomett IC](https://www.ncbi.nlm.nih.gov/pubmed/?term=Veomett%20IC%5BAuthor%5D&cauthor=true&cauthor_uid=7051833), et al. Balanced translocation karyotypes in patients with repetitive abortion. Case study and literature review. [Am J Obstet Gynecol](https://www.ncbi.nlm.nih.gov/pubmed/?term=Balanced+translocation+karyotypes+in+patients+with+repetitive+abortion) 1982;144:229-33.

[62] [Goddijn M](https://www.ncbi.nlm.nih.gov/pubmed/?term=Goddijn%20M%5BAuthor%5D&cauthor=true&cauthor_uid=14990541), [Joosten JH](https://www.ncbi.nlm.nih.gov/pubmed/?term=Joosten%20JH%5BAuthor%5D&cauthor=true&cauthor_uid=14990541), [Knegt AC](https://www.ncbi.nlm.nih.gov/pubmed/?term=Knegt%20AC%5BAuthor%5D&cauthor=true&cauthor_uid=14990541), [et](https://www.ncbi.nlm.nih.gov/pubmed/?term=Leschot%20NJ%5BAuthor%5D&cauthor=true&cauthor_uid=14990541) al. Clinical relevance of diagnosing structural chromosome abnormalities in couples with repeated miscarriage. [Hum Reprod](https://www.ncbi.nlm.nih.gov/pubmed/14990541) 2004;19:1013-7.

[63] [Stephenson MD](https://www.ncbi.nlm.nih.gov/pubmed/?term=Stephenson%20MD%5BAuthor%5D&cauthor=true&cauthor_uid=16396938), [Sierra S](https://www.ncbi.nlm.nih.gov/pubmed/?term=Sierra%20S%5BAuthor%5D&cauthor=true&cauthor_uid=16396938). Reproductive outcomes in recurrent pregnancy loss associated with a parental carrier of a structural chromosome rearrangement. [Hum Reprod](https://www.ncbi.nlm.nih.gov/pubmed/?term=Reproductive+outcomes+in+recurrent+pregnancy+loss+associated+with+a+parental+carrier+of+a+structural+chromosome+rearrangement) 2006;21:1076-82.

[64] [Brugnon F](https://www.ncbi.nlm.nih.gov/pubmed/?term=Brugnon%20F%5BAuthor%5D&cauthor=true&cauthor_uid=16339168), [Van Assche E](https://www.ncbi.nlm.nih.gov/pubmed/?term=Van%20Assche%20E%5BAuthor%5D&cauthor=true&cauthor_uid=16339168), [Verheyen G](https://www.ncbi.nlm.nih.gov/pubmed/?term=Verheyen%20G%5BAuthor%5D&cauthor=true&cauthor_uid=16339168), et al. Study of two markers of apoptosis and meiotic segregation in ejaculated sperm of chromosomal translocation carrier patients. [Hum Reprod](https://www.ncbi.nlm.nih.gov/pubmed/?term=Study+of+two+markers+of+apoptosis+and+meiotic+segregation+in+ejaculated+sperm+of+chromosomal+translocation+carrier+patients) 2006;21:685-93.

[65] [Dohle GR](https://www.ncbi.nlm.nih.gov/pubmed/?term=Dohle%20GR%5BAuthor%5D&cauthor=true&cauthor_uid=11756355), [Halley DJ](https://www.ncbi.nlm.nih.gov/pubmed/?term=Halley%20DJ%5BAuthor%5D&cauthor=true&cauthor_uid=11756355), [Van Hemel JO](https://www.ncbi.nlm.nih.gov/pubmed/?term=Van%20Hemel%20JO%5BAuthor%5D&cauthor=true&cauthor_uid=11756355), [et](https://www.ncbi.nlm.nih.gov/pubmed/?term=Govaerts%20LC%5BAuthor%5D&cauthor=true&cauthor_uid=11756355) al. Genetic risk factors in infertile men with severe oligozoospermia and azoospermia. [Hum Reprod](https://www.ncbi.nlm.nih.gov/pubmed/11756355) 2002;17:13-6.

[66] [Estop AM](https://www.ncbi.nlm.nih.gov/pubmed/?term=Estop%20AM%5BAuthor%5D&cauthor=true&cauthor_uid=7736796), [Van Kirk V](https://www.ncbi.nlm.nih.gov/pubmed/?term=Van%20Kirk%20V%5BAuthor%5D&cauthor=true&cauthor_uid=7736796), [Cieply K](https://www.ncbi.nlm.nih.gov/pubmed/?term=Cieply%20K%5BAuthor%5D&cauthor=true&cauthor_uid=7736796). Segregation analysis of four translocations, t(2;18), t(3;15), t(5;7), and t(10;12), by sperm chromosome studies and a review of the literature. [Cytogenet Cell Genet](https://www.ncbi.nlm.nih.gov/pubmed/7736796) 1995;70:80-7.

[67] [Findikli N](https://www.ncbi.nlm.nih.gov/pubmed/?term=Findikli%20N%5BAuthor%5D&cauthor=true&cauthor_uid=14680550), [Kahraman S](https://www.ncbi.nlm.nih.gov/pubmed/?term=Kahraman%20S%5BAuthor%5D&cauthor=true&cauthor_uid=14680550), [Kumtepe Y](https://www.ncbi.nlm.nih.gov/pubmed/?term=Kumtepe%20Y%5BAuthor%5D&cauthor=true&cauthor_uid=14680550), et al. Embryo development characteristics in Robertsonian and reciprocal translocations: a comparison of results with non-translocation cases. [Reprod Biomed Online](https://www.ncbi.nlm.nih.gov/pubmed/?term=Embryo+development+characteristics+in+Robertsonian+and+reciprocal+translocations%3A+a+comparison+of+results+with+non-translocation+cases) 2003;7:563-71.

[68] [Gadow EC](https://www.ncbi.nlm.nih.gov/pubmed/?term=Gadow%20EC%5BAuthor%5D&cauthor=true&cauthor_uid=1789279), [Lippold S](https://www.ncbi.nlm.nih.gov/pubmed/?term=Lippold%20S%5BAuthor%5D&cauthor=true&cauthor_uid=1789279), [Otano L](https://www.ncbi.nlm.nih.gov/pubmed/?term=Otano%20L%5BAuthor%5D&cauthor=true&cauthor_uid=1789279), et al. Chromosome rearrangements among couples with pregnancy losses and other adverse reproductive outcomes. [Am J Med Genet](https://www.ncbi.nlm.nih.gov/pubmed/?term=Chromosome+Rearrangements+Among+Couples+With+Pregnancy+Losses+and+Other+Adverse+Reproductive+Outcomes) 1991;41:279-81.

[69] [Gekas J](https://www.ncbi.nlm.nih.gov/pubmed/?term=Gekas%20J%5BAuthor%5D&cauthor=true&cauthor_uid=11139542), [Thepot F](https://www.ncbi.nlm.nih.gov/pubmed/?term=Thepot%20F%5BAuthor%5D&cauthor=true&cauthor_uid=11139542), [Turleau C](https://www.ncbi.nlm.nih.gov/pubmed/?term=Turleau%20C%5BAuthor%5D&cauthor=true&cauthor_uid=11139542), et al. Chromosomal factors of infertility in candidate couples for ICSI: an equal risk of constitutional aberrations in women and men. [Hum Reprod](https://www.ncbi.nlm.nih.gov/pubmed/?term=Gekas+J%2C+Thepot+F%2C+Turleau+C) 2001;16:82-90.

[70] [Gianaroli L](https://www.ncbi.nlm.nih.gov/pubmed/?term=Gianaroli%20L%5BAuthor%5D&cauthor=true&cauthor_uid=12456624), [Magli MC](https://www.ncbi.nlm.nih.gov/pubmed/?term=Magli%20MC%5BAuthor%5D&cauthor=true&cauthor_uid=12456624), [Ferraretti AP](https://www.ncbi.nlm.nih.gov/pubmed/?term=Ferraretti%20AP%5BAuthor%5D&cauthor=true&cauthor_uid=12456624), [et](https://www.ncbi.nlm.nih.gov/pubmed/?term=Crippa%20A%5BAuthor%5D&cauthor=true&cauthor_uid=12456624) al. Possible interchromosomal effect in embryos generated by gametes from translocation carriers. [Hum Reprod](https://www.ncbi.nlm.nih.gov/pubmed/?term=Possible+interchromosomal+effect+in+embryos+generated+by+gametes+from+translocation+carriers) 2002;17:3201-7.

[71] [Anton E](https://www.ncbi.nlm.nih.gov/pubmed/?term=Anton%20E%5BAuthor%5D&cauthor=true&cauthor_uid=18813133), [Vidal F](https://www.ncbi.nlm.nih.gov/pubmed/?term=Vidal%20F%5BAuthor%5D&cauthor=true&cauthor_uid=18813133), [Blanco J](https://www.ncbi.nlm.nih.gov/pubmed/?term=Blanco%20J%5BAuthor%5D&cauthor=true&cauthor_uid=18813133). Reciprocal translocations: tracing their meiotic behavior. [Genet Med](https://www.ncbi.nlm.nih.gov/pubmed/?term=Reciprocal+translocations%3A+tracing+their+meiotic+behavior) 2008;10:730-8.

[72] [Mierla D](https://www.ncbi.nlm.nih.gov/pubmed/?term=Mierla%20D%5BAuthor%5D&cauthor=true&cauthor_uid=24696767), [Jardan D](https://www.ncbi.nlm.nih.gov/pubmed/?term=Jardan%20D%5BAuthor%5D&cauthor=true&cauthor_uid=24696767), [Stoian V](https://www.ncbi.nlm.nih.gov/pubmed/?term=Stoian%20V%5BAuthor%5D&cauthor=true&cauthor_uid=24696767). Chromosomal abnormality in men with impaired spermatogenesis. [Int J Fertil Steril](https://www.ncbi.nlm.nih.gov/pubmed/24696767) 2014;8:35-42.

[73] [Iyer P](https://www.ncbi.nlm.nih.gov/pubmed/?term=Iyer%20P%5BAuthor%5D&cauthor=true&cauthor_uid=17359584), [Wani L](https://www.ncbi.nlm.nih.gov/pubmed/?term=Wani%20L%5BAuthor%5D&cauthor=true&cauthor_uid=17359584), [Joshi S](https://www.ncbi.nlm.nih.gov/pubmed/?term=Joshi%20S%5BAuthor%5D&cauthor=true&cauthor_uid=17359584), et al. Cytogenetic investigations in couples with repeated miscarriages and malformed children: report of a novel insertion. [Reprod Biomed Online](https://www.ncbi.nlm.nih.gov/pubmed/?term=Cytogenetic+investigations+in+couples+with+repeated+miscarriages+and+malformed+children%3A+report+of+a+novel+insertion) 2007;14:314-21.

[74] [Martin RH](https://www.ncbi.nlm.nih.gov/pubmed/?term=Martin%20RH%5BAuthor%5D&cauthor=true&cauthor_uid=8335473), [Hultén M](https://www.ncbi.nlm.nih.gov/pubmed/?term=Hult%C3%A9n%20M%5BAuthor%5D&cauthor=true&cauthor_uid=8335473). Chromosome complements in 695 sperm from three men heterozygous for reciprocal translocations, and a review of the literature. [Hereditas](https://www.ncbi.nlm.nih.gov/pubmed/?term=Chromosome+complements+in+695+sperm+from+three+men+heterozygous+for+reciprocal+translocations%2C+and+a+review+of+the+literature) 1993;118: 165-75.

[75] [Sugiura-Ogasawara M](https://www.ncbi.nlm.nih.gov/pubmed/?term=Sugiura-Ogasawara%20M%5BAuthor%5D&cauthor=true&cauthor_uid=18414779), [Aoki K](https://www.ncbi.nlm.nih.gov/pubmed/?term=Aoki%20K%5BAuthor%5D&cauthor=true&cauthor_uid=18414779), [Fujii T](https://www.ncbi.nlm.nih.gov/pubmed/?term=Fujii%20T%5BAuthor%5D&cauthor=true&cauthor_uid=18414779), et al. Subsequent pregnancy outcomes in recurrent miscarriage patients with a paternal or maternal carrier of a structural chromosome rearrangement. [J Hum Genet](https://www.ncbi.nlm.nih.gov/pubmed/18414779) 2008;53:622-8.

[76] [Portnoï MF](https://www.ncbi.nlm.nih.gov/pubmed/?term=Portno%C3%AF%20MF%5BAuthor%5D&cauthor=true&cauthor_uid=3242501), [Joye N](https://www.ncbi.nlm.nih.gov/pubmed/?term=Joye%20N%5BAuthor%5D&cauthor=true&cauthor_uid=3242501), [van den Akker J](https://www.ncbi.nlm.nih.gov/pubmed/?term=van%20den%20Akker%20J%5BAuthor%5D&cauthor=true&cauthor_uid=3242501), et al. Karyotypes of 1142 couples with recurrent abortion. [Obstet Gynecol](https://www.ncbi.nlm.nih.gov/pubmed/3242501) 1988;72:31-4.

[77] [Machev N](https://www.ncbi.nlm.nih.gov/pubmed/?term=Machev%20N%5BAuthor%5D&cauthor=true&cauthor_uid=16084877), [Gosset P](https://www.ncbi.nlm.nih.gov/pubmed/?term=Gosset%20P%5BAuthor%5D&cauthor=true&cauthor_uid=16084877), [Warter S](https://www.ncbi.nlm.nih.gov/pubmed/?term=Warter%20S%5BAuthor%5D&cauthor=true&cauthor_uid=16084877), et al. Fluorescence in situ hybridization sperm analysis of six translocation carriers provides evidence of an interchromosomal effect. [Fertil Steril](https://www.ncbi.nlm.nih.gov/pubmed/?term=Fluorescence+in+situ+hybridization+sperm+analysis+of+six+translocation+carriers+provides+evidence+of+an+interchromosomal+effect) 2005;84:365-73.

[78] [Mau UA](https://www.ncbi.nlm.nih.gov/pubmed/?term=Mau%20UA%5BAuthor%5D&cauthor=true&cauthor_uid=9194642), [Bäckert IT](https://www.ncbi.nlm.nih.gov/pubmed/?term=B%C3%A4ckert%20IT%5BAuthor%5D&cauthor=true&cauthor_uid=9194642), [Kaiser P](https://www.ncbi.nlm.nih.gov/pubmed/?term=Kaiser%20P%5BAuthor%5D&cauthor=true&cauthor_uid=9194642), [et al](https://www.ncbi.nlm.nih.gov/pubmed/?term=Kiesel%20L%5BAuthor%5D&cauthor=true&cauthor_uid=9194642). Chromosomal findings in 150 couples referred for genetic counselling prior to intracytoplasmic sperm injection. [Hum Reprod](https://www.ncbi.nlm.nih.gov/pubmed/?term=Chromosomal+findings+in+150+couples+referred+for+genetic+counselling+prior+to+intracytoplasmic+sperm+injection) 1997;12:930-7.

[79] [Meza-Espinoza JP](https://www.ncbi.nlm.nih.gov/pubmed/?term=Meza-Espinoza%20JP%5BAuthor%5D&cauthor=true&cauthor_uid=18645257), [Anguiano LO](https://www.ncbi.nlm.nih.gov/pubmed/?term=Anguiano%20LO%5BAuthor%5D&cauthor=true&cauthor_uid=18645257), [Rivera H](https://www.ncbi.nlm.nih.gov/pubmed/?term=Rivera%20H%5BAuthor%5D&cauthor=true&cauthor_uid=18645257). Chromosomal abnormalities in couples with reproductive disorders. [Gynecol Obstet Invest](https://www.ncbi.nlm.nih.gov/pubmed/18645257) 2008;66:237-40.

[80] [Ikuma S](https://www.ncbi.nlm.nih.gov/pubmed/?term=Ikuma%20S%5BAuthor%5D&cauthor=true&cauthor_uid=26083495), [Sato T](https://www.ncbi.nlm.nih.gov/pubmed/?term=Sato%20T%5BAuthor%5D&cauthor=true&cauthor_uid=26083495), [Sugiura-Ogasawara M](https://www.ncbi.nlm.nih.gov/pubmed/?term=Sugiura-Ogasawara%20M%5BAuthor%5D&cauthor=true&cauthor_uid=26083495), et al. Preimplantation Genetic Diagnosis and Natural Conception: A Comparison of Live Birth Rates in Patients with Recurrent Pregnancy Loss Associated with Translocation. [PLoS One](https://www.ncbi.nlm.nih.gov/pubmed/?term=Preimplantation+Genetic+Diagnosis+and+Natural+Conception%3A+A+Comparison+of+Live+Birth+Rates+in+Patients+with+Recurrent+Pregnancy+Loss+Associated+with+Translocation) 2015;10:e0129958.

[81] [Pundir J](https://www.ncbi.nlm.nih.gov/pubmed/?term=Pundir%20J%5BAuthor%5D&cauthor=true&cauthor_uid=27343738), [Magdalani L](https://www.ncbi.nlm.nih.gov/pubmed/?term=Magdalani%20L%5BAuthor%5D&cauthor=true&cauthor_uid=27343738), [El-Toukhy T](https://www.ncbi.nlm.nih.gov/pubmed/?term=El-Toukhy%20T%5BAuthor%5D&cauthor=true&cauthor_uid=27343738). Outcome of preimplantation genetic diagnosis using FISH analysis for recurrent miscarriage in low-risk reciprocal translocation carriers. [Eur J Obstet Gynecol Reprod Biol](https://www.ncbi.nlm.nih.gov/pubmed/?term=Outcome+of+preimplantation+genetic+diagnosis+using+FISH+analysis+for+recurrent+miscarriage+in+low-risk+reciprocal+translocation+carriers) 2016;203:214-9.

[82] [Schwartz S](https://www.ncbi.nlm.nih.gov/pubmed/?term=Schwartz%20S%5BAuthor%5D&cauthor=true&cauthor_uid=6832778), [Palmer CG](https://www.ncbi.nlm.nih.gov/pubmed/?term=Palmer%20CG%5BAuthor%5D&cauthor=true&cauthor_uid=6832778). Chromosomal findings in 164 couples with repeated spontaneous abortions: with special consideration to prior reproductive history. [Hum Genet](https://www.ncbi.nlm.nih.gov/pubmed/?term=Chromosomal+Findings+in+164+Couples+with+Repeated+Spontaneous+Abortions%3A+with+Special+Consideration+to+Prior+Reproductive+History) 1983;63:28-34.

[83] [Peschka B](https://www.ncbi.nlm.nih.gov/pubmed/?term=Peschka%20B%5BAuthor%5D&cauthor=true&cauthor_uid=10469691), [Leygraaf J](https://www.ncbi.nlm.nih.gov/pubmed/?term=Leygraaf%20J%5BAuthor%5D&cauthor=true&cauthor_uid=10469691), [Van der Ven K](https://www.ncbi.nlm.nih.gov/pubmed/?term=Van%20der%20Ven%20K%5BAuthor%5D&cauthor=true&cauthor_uid=10469691), et al. Type and frequency of chromosome aberrations in 781 couples undergoing intracytoplasmic sperm injection. [Hum Reprod](https://www.ncbi.nlm.nih.gov/pubmed/?term=Type+and+frequency+of+chromosome+aberrations+in+781+couples+undergoing+intracytoplasmic+sperm+injection) 1999;14:2257-63.

[84] [Douet-Guilbert N](https://www.ncbi.nlm.nih.gov/pubmed/?term=Douet-Guilbert%20N%5BAuthor%5D&cauthor=true&cauthor_uid=16300670), [Bris MJ](https://www.ncbi.nlm.nih.gov/pubmed/?term=Bris%20MJ%5BAuthor%5D&cauthor=true&cauthor_uid=16300670), [Amice V](https://www.ncbi.nlm.nih.gov/pubmed/?term=Amice%20V%5BAuthor%5D&cauthor=true&cauthor_uid=16300670), et al. Interchromosomal effect in sperm of males with translocations: report of 6 cases and review of the literature. [Int J Androl](https://www.ncbi.nlm.nih.gov/pubmed/?term=Interchromosomal+effect+in+sperm+of+males+with+translocations%3A+report+of+6+cases+and+review+of+the+literature) 2005;28:372-9.

[85] [Blanco J](https://www.ncbi.nlm.nih.gov/pubmed/?term=Blanco%20J%5BAuthor%5D&cauthor=true&cauthor_uid=10914679), [Egozcue J](https://www.ncbi.nlm.nih.gov/pubmed/?term=Egozcue%20J%5BAuthor%5D&cauthor=true&cauthor_uid=10914679), [Vidal F](https://www.ncbi.nlm.nih.gov/pubmed/?term=Vidal%20F%5BAuthor%5D&cauthor=true&cauthor_uid=10914679). Interchromosomal effects for chromosome 21 in carriers of structural chromosome reorganizations determined by fluorescence in situ hybridization on sperm nuclei. [Hum Genet](https://www.ncbi.nlm.nih.gov/pubmed/?term=Interchromosomal+effect+for+chromosome+21+in+carriers+of+structural+chromosome+reorganizations+determined+by+fluorescence+in+situ+hybridization+on+sperm+nuclei.) 2000;106:500-5.

[86] [Pellestor F](https://www.ncbi.nlm.nih.gov/pubmed/?term=Pellestor%20F%5BAuthor%5D&cauthor=true&cauthor_uid=11387286), [Imbert I](https://www.ncbi.nlm.nih.gov/pubmed/?term=Imbert%20I%5BAuthor%5D&cauthor=true&cauthor_uid=11387286), [Andréo B](https://www.ncbi.nlm.nih.gov/pubmed/?term=Andr%C3%A9o%20B%5BAuthor%5D&cauthor=true&cauthor_uid=11387286), [et](https://www.ncbi.nlm.nih.gov/pubmed/?term=Lefort%20G%5BAuthor%5D&cauthor=true&cauthor_uid=11387286) al. Study of the occurrence of interchromosomal effect in spermatozoa of chromosomal rearrangement carriers by fluorescence in-situ hybridization and primed in-situ labelling techniques. [Hum Reprod](https://www.ncbi.nlm.nih.gov/pubmed/?term=Study+of+the+occurrence+of+interchromosomal+effect+in+spermatozoa+of+chromosomal+rearrangement+carriers+by+fluorescence+in-situ+hybridization+and+primed+in-situ+labelling+techniques.) 2001;16:1155-64.

[87] [Oliver-Bonet M](https://www.ncbi.nlm.nih.gov/pubmed/?term=Oliver-Bonet%20M%5BAuthor%5D&cauthor=true&cauthor_uid=12356948), [Navarro J](https://www.ncbi.nlm.nih.gov/pubmed/?term=Navarro%20J%5BAuthor%5D&cauthor=true&cauthor_uid=12356948), [Carrera M](https://www.ncbi.nlm.nih.gov/pubmed/?term=Carrera%20M%5BAuthor%5D&cauthor=true&cauthor_uid=12356948), et al. Aneuploid and unbalanced sperm in two translocation carriers: evaluation of the genetic risk. [Mol Hum Reprod](https://www.ncbi.nlm.nih.gov/pubmed/?term=Aneuploid+and+unbalanced+sperm+in+two+translocation+carriers%3A+evaluation+of+the+genetic+risk.) 2002;8:958-63.

[88] [Martin RH](https://www.ncbi.nlm.nih.gov/pubmed/?term=Martin%20RH%5BAuthor%5D&cauthor=true&cauthor_uid=2253936), [Barclay L](https://www.ncbi.nlm.nih.gov/pubmed/?term=Barclay%20L%5BAuthor%5D&cauthor=true&cauthor_uid=2253936), [Hildebrand K](https://www.ncbi.nlm.nih.gov/pubmed/?term=Hildebrand%20K%5BAuthor%5D&cauthor=true&cauthor_uid=2253936), et al. Cytogenetic analysis of 400 sperm from three translocation heterozygotes. [Hum Genet](https://www.ncbi.nlm.nih.gov/pubmed/?term=Cytogenetic+analysis+of+400+sperm+from+three+translocation+heterozygotes.) 1990;86:33-9.

[89] [Martin RH](https://www.ncbi.nlm.nih.gov/pubmed/?term=Martin%20RH%5BAuthor%5D&cauthor=true&cauthor_uid=7774043), [Spriggs EL](https://www.ncbi.nlm.nih.gov/pubmed/?term=Spriggs%20EL%5BAuthor%5D&cauthor=true&cauthor_uid=7774043). Sperm chromosome complements in a man heterozygous for a reciprocal translocation 46,XY,t(9;13)(q21.1;q21.2) and a review of the literature. [Clin Genet](https://www.ncbi.nlm.nih.gov/pubmed/?term=Sperm+chromosome+complements+in+a+man+heterozygous+for+a+reciprocal+translocation+46%2CXY%2Ct(9%3B13)(q21.1%3Bq21.1)+and+a+review+of+the+literature.) 1995;47:42-6.

[90] [Oliver-Bonet M](https://www.ncbi.nlm.nih.gov/pubmed/?term=Oliver-Bonet%20M%5BAuthor%5D&cauthor=true&cauthor_uid=15333594), [Navarro J](https://www.ncbi.nlm.nih.gov/pubmed/?term=Navarro%20J%5BAuthor%5D&cauthor=true&cauthor_uid=15333594), [Codina-Pascual M](https://www.ncbi.nlm.nih.gov/pubmed/?term=Codina-Pascual%20M%5BAuthor%5D&cauthor=true&cauthor_uid=15333594), [et](https://www.ncbi.nlm.nih.gov/pubmed/?term=Benet%20J%5BAuthor%5D&cauthor=true&cauthor_uid=15333594) al. From spermatocytes to sperm: meiotic behaviour of human male reciprocal translocations. [Hum Reprod](https://www.ncbi.nlm.nih.gov/pubmed/15333594) 2004;19:2515-22.

[91] [Cora T](https://www.ncbi.nlm.nih.gov/pubmed/?term=Cora%20T%5BAuthor%5D&cauthor=true&cauthor_uid=12399524), [Acar H](https://www.ncbi.nlm.nih.gov/pubmed/?term=Acar%20H%5BAuthor%5D&cauthor=true&cauthor_uid=12399524), [Kaynak M](https://www.ncbi.nlm.nih.gov/pubmed/?term=Kaynak%20M%5BAuthor%5D&cauthor=true&cauthor_uid=12399524). Molecular cytogenetic detection of meiotic segregation patterns in sperm nuclei of carriers of 46,XY,t(15;17)(q21; q25). [J Androl](https://www.ncbi.nlm.nih.gov/pubmed/?term=Molecular+cytogenetic+detection+of+meiotic+segregation+patterns+in+sperm+nuclei+of+carriers+of+46%2CXY%2Ct(15%3B17)(q21%3Bq25).) 2002;23:793-8.

[92] [Goldman AS](https://www.ncbi.nlm.nih.gov/pubmed/?term=Goldman%20AS%5BAuthor%5D&cauthor=true&cauthor_uid=8432191), [Hultén MA](https://www.ncbi.nlm.nih.gov/pubmed/?term=Hult%C3%A9n%20MA%5BAuthor%5D&cauthor=true&cauthor_uid=8432191). Meiotic analysis by FISH of a human male 46,XY,t(15;20)(q11.2;q11.2) translocation heterozygote: quadrivalent configuration, orientation and first meiotic segregation. [Chromosoma](https://www.ncbi.nlm.nih.gov/pubmed/?term=Meiotic+analysis+by+FISH+of+a+human+male+46%2CXY%2Ct(15%3B20)(q11.2%3Bq11.2)+translocation+heterozygote%3A+quadrivalent+configuration%2C+orientation+and+first+meiotic+segregation.) 1993;102:102-11.

Supplementary Table 2. Incidence of breakpoints on group D chromosomes

| Breakpoints | Number of patients with pre-gestational infertility | Number of patients with gestational infertility | Total |
| --- | --- | --- | --- |
| 13p13 | 2 |  | 2 |
| 13p12 | 1 |  | 1 |
| 13p11.2 | 3 |  | 3 |
| 13p11 | 2 |  | 2 |
| 13q10 | 3 | 1 | 4 |
| 13q11 | 2 |  | 2 |
| 13q12 | 4 | 2 | 6 |
| 13q12.1 |  | 1 | 1 |
| 13q12.3 | 1 |  | 1 |
| 13q13 | 2 | 3 | 5 |
| 13q14 | 5 | 2 | 7 |
| 13q14.1 |  | 5 | 5 |
| 13q14.2 |  | 2 | 2 |
| 13q14.3 |  | 2 | 2 |
| 13q15 | 1 |  | 1 |
| 13q21 |  | 4 | 4 |
| 13q21.1 |  | 2 | 2 |
| 13q21.2 |  | 3 | 3 |
| 13q21.3 | 1 |  | 1 |
| 13q22 | 2 | 10 | 12 |
| 13q22.3 |  | 2 | 2 |
| 13q24 |  | 1 | 1 |
| 13q31 | 1 | 4 | 5 |
| 13q31.2 |  | 1 | 1 |
| 13q32 | 1 | 6 | 7 |
| 13q32.2 |  | 1 | 1 |
| 13q33 |  | 3 | 3 |
| 13q33.1 |  | 1 | 1 |
| 13q34 | 1 | 3 | 4 |
| 13qter |  | 1 | 1 |
| 14p12 | 1 |  | 1 |
| 14p11.2 | 2 | 2 | 4 |
| 14p10 | 3 |  | 3 |
| 14q11 | 1 | 3 | 4 |
| 14q11.2 | 2 | 1 | 3 |
| 14q12 | 1 | 2 | 3 |
| 14q13 | 1 | 2 | 3 |
| 14q13.3 |  | 1 | 1 |
| 14q21 | 2 | 1 | 3 |
| 14q22 | 1 | 5 | 6 |
| 14q22.3 |  | 1 | 1 |
| 14q23 |  | 1 | 1 |
| 14q24 | 2 | 4 | 6 |
| 14q24.1 | 1 |  | 1 |
| 14q24.2 |  | 1 | 1 |
| 14q24.3 |  | 1 | 1 |
| 14q31 | 2 | 1 | 3 |
| 14q32 |  | 11 | 11 |
| 14q32.1 |  | 2 | 2 |
| 14q32.2 |  | 2 | 2 |
| 14q32.3 |  | 2 | 2 |
| 15p13 | 1 |  | 1 |
| 15p12 | 2 | 2 | 4 |
| 15p11.2 | 1 | 1 | 2 |
| 15p11.1 |  | 1 | 1 |
| 15p11 | 2 | 1 | 3 |
| 15p10 | 1 |  | 1 |
| 15q11 | 3 | 1 | 4 |
| 15q11.2 |  | 2 | 2 |
| 15q12 | 1 | 1 | 2 |
| 15q13 | 1 | 1 | 2 |
| 15q14 |  | 1 | 1 |
| 15q15 | 7 | 2 | 9 |
| 15q15.3 |  | 1 | 1 |
| 15q21 |  | 4 | 4 |
| 15q21.1 |  | 2 | 2 |
| 15q22 |  | 9 | 9 |
| 15q22.2 | 1 |  | 1 |
| 15q23 |  | 1 | 1 |
| 15q24 | 2 | 5 | 7 |
| 15q25 |  | 2 | 2 |
| 15q26 |  | 3 | 3 |
| 15q26.1 |  | 5 | 5 |
| 15q26.2 |  | 3 | 3 |
| 15q26.3 | 1 | 3 | 4 |
